# SINEUP long non-coding RNA acts via PTBP1 and HNRNPK to promote translational initiation assemblies

**DOI:** 10.1093/nar/gkaa814

**Published:** 2020-11-02

**Authors:** Naoko Toki, Hazuki Takahashi, Harshita Sharma, Matthew N Z Valentine, Ferdous-Ur M Rahman, Silvia Zucchelli, Stefano Gustincich, Piero Carninci

**Affiliations:** Laboratory for Transcriptome Technology, RIKEN Center for Integrative Medical Sciences, Yokohama, Kanagawa 230-0045 Japan; Functional Genomics Laboratory, Graduate School of Medical Life Science, Yokohama City University, Yokohama, Japan; Laboratory for Transcriptome Technology, RIKEN Center for Integrative Medical Sciences, Yokohama, Kanagawa 230-0045 Japan; Functional Genomics Laboratory, Graduate School of Medical Life Science, Yokohama City University, Yokohama, Japan; Laboratory for Transcriptome Technology, RIKEN Center for Integrative Medical Sciences, Yokohama, Kanagawa 230-0045 Japan; Laboratory for Transcriptome Technology, RIKEN Center for Integrative Medical Sciences, Yokohama, Kanagawa 230-0045 Japan; Laboratory for Transcriptome Technology, RIKEN Center for Integrative Medical Sciences, Yokohama, Kanagawa 230-0045 Japan; Department of Health Sciences, Center for Autoimmune and Allergic Diseases (CAAD) and Interdisciplinary Research Center of Autoimmune Diseases (IRCAD), University of Piemonte Orientale, Novara, Italy; Department of Neuroscience and Brain Technologies, Istituto Italiano di Tecnologia, Genova, Italy; Laboratory for Transcriptome Technology, RIKEN Center for Integrative Medical Sciences, Yokohama, Kanagawa 230-0045 Japan; Functional Genomics Laboratory, Graduate School of Medical Life Science, Yokohama City University, Yokohama, Japan

## Abstract

SINEUPs are long non-coding RNAs (lncRNAs) that contain a SINE element, and which up-regulate the translation of target mRNA. They have been studied in a wide range of applications, as both biological and therapeutic tools, although the underpinning molecular mechanism is unclear. Here, we focused on the sub-cellular distribution of target mRNAs and *SINEUP* RNAs, performing co-transfection of expression vectors for these transcripts into human embryonic kidney cells (HEK293T/17), to investigate the network of translational regulation. The results showed that co-localization of target mRNAs and *SINEUP* RNAs in the cytoplasm was a key phenomenon. We identified PTBP1 and HNRNPK as essential RNA binding proteins. These proteins contributed to *SINEUP* RNA sub-cellular distribution and to assembly of translational initiation complexes, leading to enhanced target mRNA translation. These findings will promote a better understanding of the mechanisms employed by regulatory RNAs implicated in efficient protein translation.

## INTRODUCTION

More than 70% of the mammalian genome is transcribed into RNAs (1,2). The majority of these RNAs do not encode proteins and are called non-coding RNAs. A large fraction of non-coding RNAs are found as sense-antisense pairwise transcripts that are co-regulated at the transcriptional level (3). Although some of these non-coding RNAs, such as transfer RNA (tRNA) and ribosomal RNA (rRNA), have been intensely studied, the biological function of 98.5% of long non-coding RNAs (lncRNAs), which are defined as non-coding RNAs longer than 200 nucleotides (4), are still unknown (5). Importantly, some lncRNAs have regulatory functions at the transcriptional or translational levels (6–8).

We previously found that AS-*Uchl1*, a natural antisense (AS) lncRNA, was able to enhance the translation of its sense *Uchl1* (ubiquitin carboxyterminal hydrolase L1) mRNA in mouse dopaminergic cells (9). The functional element embedded in AS-*Uchl1* is an inverted SINEB2 (Short Interspersed Nuclear Element B2) that acts as an effector domain (ED) and is essential for up-regulating protein synthesis (10). This element enhanced the UCHL1 protein level without changing *Uchl1* mRNA quantities. In mouse dopaminergic neurons, mature *Uchl1* mRNA is predominantly localized in the cytoplasm, whereas natural AS-*Uchl1* RNA is localized in the nucleus at steady state. Interestingly, in rapamycin-induced stress conditions, endogenous AS-*Uchl1* RNA is transported to the cytoplasm, acting to increase *Uchl1* mRNA association to heavy polysomes and enhance its translation (9).

We also found that other natural antisense lncRNAs containing SINEs specifically up-regulate translation of partially overlapping sense mRNAs, and termed this functional class of lncRNAs ‘SINEUPs’ (10). In addition to the inverted SINEB2, SINEUPs also require an antisense sequence, known as a binding domain (BD), which overlaps the target sense mRNA (10). Importantly, the SINEUPs have been shown to function broadly in different cell lines and organisms (10–12). In this study, we used a synthetic SINEUP targeting green fluorescent protein (*SINEUP-GFP*) to shed light on the mechanism behind the translation enhancement of target mRNAs by synthetic SINEUPs, with a specific focus on RNA subcellular distribution and its contribution to protein translation.

Here, we show that *SINEUP* RNA interacts with nucleocytoplasmic proteins such as PTBP1 (polypyrimidine tract binding protein-1) and HNRNPK (heterogeneous nuclear ribonucleoprotein K) that are essential for RNA localization and translational initiation assembly.

## MATERIALS AND METHODS

### Cell culture

Human Embryonic Kidney (HEK) 293T/17 cells purchased from ATCC were maintained in DMEM, high glucose, GlutaMAX™ Supplement, pyruvate (Gibco) supplemented with 10% fetal bovine serum (Sigma) and 1% penicillin–streptomycin (Wako) at 37°C, 5% CO_2_.

### Plasmid and constructs

The pEGFP-C2 vector (expression vector for EGFP) was purchased from Clontech. SINEUP-GFP in pcDNA3.1 (−) vector was described previously in Carrieri *et al.* (9). SINEUP-SCR, SINEUP-ΔSB2, SINEUP-ΔAlu and SINEUP-GFP constructs were cloned into the pCS2+ vector (12). SINEUP-ΔPTBP1 binding regions (Δ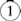-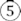) and SINEUP-ΔHNRNPK binding regions (Δ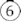-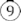) were designed with deletion of the binding sites from SINEUP-GFP. The BD (−34/+10) of SINEUP-UCHL1 is designed to replace the BD of SINEUP-GFP.

### Plasmid transfection and conditions

The pEGFP-C2 and SINEUP vectors were co-transfected into HEK293T/17 cells by using Lipofectamine 2000 (Invitrogen) with OptiMEM (1×) Reduced Serum Medium (Gibco). Testing *EGFP* mRNA translation at various time points after transfection confirmed that up-regulation of translation of *EGFP* mRNA occurred from 24 to 48 h post-transfection. SINEUP-UCHL1 vector was transfected in HEK293T/17 cells as mentioned above and harvested after 48 h of vector transfection.

### Measuring protein up-regulation by Western blotting assay

Cells were plated in twelve-well plates, transfected with plasmid(s), lysed in Cell Lysis buffer (Cell Signaling), and incubated at 4°C for 1 h. The cell lysates were applied to 10% precast polyacrylamide gels (Bio-Rad) for SDS-PAGE and transferred to nitrocellulose membranes (Amersham). The membranes were incubated for 1 h at room temperature with the primary antibody, anti-GFP rabbit polyclonal antibody (1:1000 dilution; A6455, Thermo Fisher Scientific), and then for 45 min at room temperature with the secondary antibody, anti-rabbit IgG conjugated with HRP (P0448, Dako), and EGFP was detected by ECL detection reagent (Amersham). As a control, anti-β actin mouse monoclonal antibody (1:1000 dilution; A5441, Sigma Aldrich) was used as the primary antibody, and anti-mouse IgG–conjugated HRP (1:1000 dilution; P0447, Dako) was used as the secondary antibody. To detect endogenous target proteins, anti-hnRNPK mouse monoclonal antibody [3C2]-ChIP Grade (ab39975, Abcam), anti-PTBP1 mouse monoclonal antibody (32-4800, Thermo Fisher Scientific), RPL7A rabbit polyclonal antibody (PA5-30155, Thermo Fisher Scientific), RPS3A rabbit polyclonal antibody (PA5-29398, Thermo Fisher Scientific), and anti-UCHL1 mouse monoclonal antibody (CL3210, Sigma Aldrich) were used at 1:1000 dilution with overnight incubation at 4°C.

### RNA extraction and reverse transcription real-time quantitative PCR (RT-qPCR)

RNA was extracted using the RNeasy mini kit (QIAGEN) following the manufacturer's instructions. For separation of nuclear and cytoplasmic fractions, the PARIS kit (ThermoFisher) was used to obtain nucleic and cytoplasmic fractionated lysate. TURBO DNA-free Kit (Invitrogen) was used for DNase I treatment to remove plasmid DNAs. For RT-qPCR, cDNA was synthesized using the PrimeScript 1st strand cDNA synthesis kit (TAKARA), and PCR was performed with SYBR Premix Ex *Taq* II (TAKARA) and the 7900HT Fast Real-Time PCR System (Applied Biosystems). The thermocycling protocol was 95°C for 30 s followed by 40 cycles of 95°C for 5 s and 60°C for 30 s. The RNA expression level was normalized to the level of *GAPDH* mRNA in each fraction. The primer sequence of *SINEUP* RNAs and *GFP* mRNAs was described in published paper (10). *UCHL1* mRNA was amplified using the forward primer, 5′-CCTGAAGACAGAGCAAAATGC-3′, and the revers primer, 5′-TGAATTCTCTGCAGACCTTGG-3′.

### RNA FISH

FISH probes for target transcripts were designed using Stellaris RNA FISH designer (BIOSEARCH Technologies; https://www.biosearchtech.com/support/tools/design-software/stellaris-probe-designer), and fluorescently labelled with Quasar 570 (for *SINEUP* RNAs) or Quasar 670 (for *EGFP* mRNA and *UCHL1* mRNA) (Supplementary Table S2). Stellaris FISH Probes, Human GAPDH with Quasar 570 Dye (SMF-2026-1, BIOSERCH) were used as a positive control for FISH assessment. Cells were fixed with 4% paraformaldehyde (WAKO) and permeabilized with 0.5% Triton X-100 (Sigma) at room temperature for 5 min. Hybridization was performed overnight at 37°C. Nuclei were visualized by incubation with Hoechst 33342 (H3570, Thermo Fisher Scientific). After sequential washing steps, cell images were detected using a SP8-HyVolution confocal laser scanning microscope (Leica Microsystems) with a 63x/1.4 oil objective lens; the images were processed using HyD detectors with Huygens Essential software (Scientific Volume Imaging). The RNA signals in the images were counted with Icy Spot Detector (http://icy.bioimageanalysis.org/plugin/Spot_Detector; (13), and percentage co-localization was calculated with Icy Colocalization Studio (http://icy.bioimageanalysis.org/plugin/Colocalization_Studio; (14).

### Detection of SINEUP RBPs

The protocol for detecting SINEUP RBPs was based on the protocol for the Magna ChIRP RNA Interactome kit (Merck Millipore) with the modification of cross-linking with 300 mJ/cm^2^ of 254 nm UV light (CL-1000 Ultraviolet Cross Link, UVP). The cell pellet (2 × 10^7^ cells) was suspended with 2 ml of Lysis buffer (Cell Signaling) supplemented with Protease Inhibitor Cocktail Set III (Merck Millipore), mixed by rotation at 4°C for 30 min, and then sonicated for 8 cycles (ON for 30 s, OFF for 30 s) using a Picoruptor Sonicator (Diagenode). Each tube of lysate was incubated at 37°C for 30 min with MagCapture Tamavidin 2-REV magnetic beads (WAKO) to remove nonspecific binding proteins, which improves specificity of capture of specific RBPs. The lysate was incubated overnight at 37°C in hybridization buffer (750 mM NaCl, 50 mM Tris–HCl (pH 7.5), 1 mM EDTA) with additional 15% (v/v) formamide (Sigma), Phenylmethanesulfonyl Fluoride (PMSF) (Cell Signaling), Protease Inhibitor Cocktail Set III (Merck Millipore) and SUPERase• In RNase Inhibitor (Thermo Fisher Scientific) just before use, and 100 pmol of probe. Each lysate was then incubated at 37°C for 30 min with washed beads. After sequential washes, the bead samples were separated into two halves for protein and RNA extraction. Proteins were extracted as reported by Chu *et al.* 2015 (15); the beads samples were re-suspended with biotin elution buffer followed by series of incubations at room temperature for 20 min and at 65°C for 10 min, and then, the eluent was incubated in DNase/RNase solution (100 μg/ml RNase A, 0.1 U/μL RNase H, and 100 U/mL DNase I) at 37°C for 1 h followed by acetone precipitation. The protein samples were digested with 10 ng/μl Sequencing Grade Modified Trypsin (V5111, Promega) overnight, and the resultant peptides were subjected to liquid chromatography–tandem mass spectrometry (LC–MS/MS) at the Support Unit for Bio-Material Analysis, Research Resource Center, Brain Science Institute in Wako, Japan. Proteome Discover (version 1.4, Thermo Fisher Scientific) software with the MASCOT search engine (version 2.6.0, Matrix Science Limited) was used in the Swiss-Prot database. For RNA extraction, the beads were incubated with proteinase K at 55°C overnight, and then extracted with Trizol (Thermo Fisher Scientific) and chloroform (WAKO). The eluent was treated with DNase I (Ambion), and the RNA expression level was quantified by RT-qPCR.

### Validation of SINEUP RBPs by siRNA-mediated knockdown

All siRNAs listed below were purchased from Thermo Fisher Scientific. DNAJC1 Silencer Select Pre-designed siRNA (ID: s34557), EEF1A1 Silencer Select Pre-designed siRNA (ID: s4479), EEF2 Silencer Select Pre-designed siRNA (ID: s4492), HNRNPK Silencer Select siRNA: Standard (ID: s6739 was used in main figures and ID: s6738 and ID: s6737 were used in Supplementary Figure S7), HNRNPM Silencer Select Pre-designed siRNA (ID: s9259), HNRNPU Silencer Select Pre-designed siRNA (ID: s6743), LMNB1 Silencer Select Pre-designed siRNA (ID: s8226), and PTBP1 Silencer Select Validated siRNA (siRNA ID: s11434 was used in main figures, and ID: s11435 and ID: s11436 were used in Supplementary Figure S7) were used for the knockdown experiments; 4390843 Silencer Select Negative Control #1 siRNA was used as the negative control. Twenty-four hours after the cells were plated, the target pre-designed siRNA was transfected by using Lipofectamine RNAiMAX (Invitrogen), and the cells were maintained in DMEM (1×) + GlutaMAX-1 (Gibco) supplemented with 10% fetal bovine serum (Sigma) without penicillin-streptomycin (Wako) at 37°C, 5% CO_2_ for 24 h. Following this, the vectors were transfected into the cells as described above. Targeted proteins were detected by using the following anti-mouse monoclonal antibodies purchased from Santa Cruz: DnaJC1[D-10] (sc-514244), EF-1 α1 [CBP-KK1] (sc-21758), EF-2 [C-9] (sc-166415), hnRNP K/J [3C2] (sc-32307), hnRNP M [A-12] (sc-515008), hnRNP I [SH54] (sc-56701), hnRNP U [3G6] (sc-32315) and Lamin B1 [8D1] (sc-56144). The above primary antibodies were diluted 1 in 500 and then incubated at 4°C overnight. HRP-conjugated anti-mouse IgG (P0447, Dako) was used as secondary antibody diluted 1 in 1000 and then incubated at room temperature for 45 min for protein visualization.

### RIP with SINEUP RBPs

RIP was performed using the Abcam protocol (https://www.abcam.com/epigenetics/rna-immunoprecipitation-rip-protocol) with some modifications. HEK293T/17 cells were plated into 10-cm plates, followed by plasmid transfection described above. On the following day, the cells were irradiated with 300 mJ/cm^2^ of 254 nm UV light, and nuclei and cytoplasmic fractions were isolated. Nuclear pellets were sheared by sonication with 5 cycles (ON for 30 s, OFF for 30 s) using a Bioruptor Pico device. To immunoprecipitate RNA with the antibodies for target proteins, each lysate was incubated at 4°C overnight. Protein A/G magnetic beads (Invitrogen) were added to bind the target antibodies, and sequential washing was conducted to remove unbound antibodies. The anti-hnRNP K mouse monoclonal antibody [3C2]-ChIP Grade (ab39975, Abcam) and anti-PTBP1 mouse monoclonal antibody (32-4800, Thermo Fisher Scientific) were used to immunoprecipitate HNRNPK and PTBP1, respectively, in each nuclear or cytoplasmic fraction. To purify the RNA, the lysates were incubated with protease K at 55°C overnight, followed by Trizol (Thermo Fisher Scientific) and chloroform (WAKO) extraction. RNA levels were quantified by RT-qPCR.

### Clone overexpression of SINEUP RBPs

Clone vectors, hnRNPK in pCMV6-XL5, and PTBP1 in pCMV6-AC, were purchased from ORIGENE. After the cells were plated for 18 h, target protein clone vectors were transfected using Lipofectamine 2000 (Invitrogen). The transfected cells were maintained in DMEM (1×) + GlutaMAX-1 (Gibco) supplemented with 10% fetal bovine serum (Sigma) without penicillin-streptomycin (Wako) at 37°C, 5% CO_2_ for 6 h. Following this, pEGFP-C2 and SINEUP vectors were transfected into the cells as described above.

### Immunofluorescence microscopy

HEK293T/17 were prepared as described above for RNA FISH. After the cells were permeabilized, the primary antibodies, anti-hnRNP K mouse monoclonal antibody [3C2]-ChIP Grade (ab39975, Abcam) and anti-PTBP1 mouse monoclonal antibody (32-4800, Thermo Fisher Scientific), were added and hybridized overnight. Alexa Fluor 647–conjugated goat anti-mouse IgG secondary antibodies (A-21236, Thermo Fisher Scientific) were used to visualize the results. Several organelle marker antibodies were tested as follows; anti-SC35 (S4045, Sigma-Aldrich), anti-nmt55/p54nrb (NONO, ab70335, Abcam), anti-ILF3 (ab133354, Abcam), anti-EF1A (sc-21758, SantaCruz), anti-ATP5 (mitochondria, ab14748, Abcam), anti-Calnexin (endoplasmic reticulum, ab202572, Abcam) and J2 monoclonal antibody (dsRNA, 10010200, SCICONS). MALAT1 probes labeled with Atto 633 (Supplementary Table S2).

### Polysome fractionation

Polysome fractionation was performed as reported previously (16). Briefly, 2.5 × 10^6^ cells were plated into 10-cm plates, followed by hnRNPK and PTBP1 clone vector transfection after 18 h, and EGFP and SINEUP vector transfection 6 h later. All transfections were performed as described above. The transfected cells were maintained in growth media without antibiotics for 48 h following clone vector transfection, then incubated with 0.1 mg/ml cycloheximide for 5 min at 37°C followed by washing with ice-cold PBS containing 0.1 mg/ml cycloheximide. The harvested cells were centrifuged at 300 × *g* for 10 min at 4°C. The cell pellets were suspended with 200 μL of ice-cold lysis buffer (50 mM Tris–HCl pH 7.5, 100 mM NaCl, 30 mM MgCl_2_, 0.1 mg/ml cycloheximide, 0.1% NP-40, with fresh RNase inhibitor and Proteinase inhibitor cocktail added just before use). The cell lysate was incubated for 10 min on ice followed by centrifugation at 2000 × *g* at 4°C for 5 min to separate the nuclei. The cytoplasmic fraction was subjected to further centrifugation at 17,000 × *g* at 4°C for 5 min to remove cell debris. The cytoplasmic lysate was layered onto a 15–45% sucrose gradient and centrifuged in an SW41Ti Beckman rotor at 190 000 × *g* at 4°C for 3.5 h. The sucrose gradient was separated into 12 fractions calculated by Triax flow cell (Biocomp). Half of each fraction was treated with Proteinase K at 55°C overnight then followed by Trizol and chloroform extractions as described above. The eluent was treated with DNase I (Ambion), and the RNA expression level was quantified by RT-qPCR.

Using the other half of each fraction, proteins were isolated using the Thermo Fisher Scientific acetone precipitation protocol, 4 volumes of cold acetone were added to each sample, and then the samples were incubated at −20°C overnight, and centrifuged at 13 000 × *g* for 10 min at 4°C. Each pellet was suspended in PBS and subjected to Western blotting analysis.

### Protein-protein direct interaction with chemical cross-linking

BS3 (bis [sulfosuccinimidyl] suberate) cross-linking was performed based on a published protocol (17). The cells were incubated with 0.6 mM BS3 (Thermo Fisher Scientific) for 30 min; the reaction was quenched by incubation with 1 M Tris–HCl (pH 7.5) for 15 min at room temperature. The cell lysate was incubated with target antibodies as described for RIP. After sequential washes, the beads were incubated with 2× Laemmli sample buffer (Bio-Rad) at 95°C for 20 min to dissociate proteins, followed by Western blotting assay.

### Double strand RNA (dsRNA) immunoprecipitation with chemical cross-linking

Formaldehyde cross-linking was performed based on the Abcam X-ChIP protocol (https://www.abcam.co.jp/protocols/cross-linking-chromatin-immunoprecipitation-x-chip-protocol), and nuclei and cytoplasmic fractions were isolated using the PARIS kit (ThermoFisher). To immunoprecipitate dsRNA with J2 monoclonal antibody (10010200, SCICONS), fractionated lysates were incubated at 4°C overnight. The beads were washed three times with 1× RIPA buffer at 37°C for 5 min, and nucleic fraction beads underwent two further DNase treatments incubated at 37°C for 30 min. RNA was extracted as mentioned in the RIP protocol and quantified by RT-qPCR.

### Detection of PTBP1 and HNRNPK binding regions on the *SINEUP-GFP* RNA

The seCLIP protocol described by Van Nostrand *et al.* (18) was performed to identify the binding regions of PTBP1 and HNRNPK on *SINEUP-GFP* RNA. Briefly, HEK293T/17 cells were plated into 10-cm plates, followed by plasmid transfection as described above. After 24 h of transfection, the cells were irradiated with 400 mJ/cm^2^ of 254 nm UV light. Next, for each sample, 2 × 10^7^ cells were lysed and treated with DNase and RNase I. Meanwhile, 10 μg anti-hnRNPK (ab39975, Abcam) or anti-PTBP1 (32-4800, Thermofisher) antibodies were coupled to 125 μl magnetic beads (Dynabeads M-280 Sheep Anti-mouse IgG). To capture RBP-RNA complexes on beads, the cell lysate was incubated with a magnetic beads-coupled antibody at 4°C overnight. Next, 2% input was saved and RNA in IP samples was dephosphorylated and end treated with Poly Nucleotide Kinase followed by on-beads 3′ RNA linker (with sample barcodes, see sequence in Supplementary Table S3) ligation. After this, RBP-RNA complexes were detected by Western blotting and the region above 55 kDa was cut out from the nitrocellulose membrane blot. In order to isolate the RNA, membrane slices were treated with Proteinase K and Urea followed by acid phenol–chloroform extraction and cleanup by Zymo RNA kit (R1013). Further treatment of input and IP samples and cDNA library preparation steps are as described in the original protocol. Finally, the amplified library was purified using AMPure XP beads in 1:1.5 ratio and quantified using qPCR and Bioanalyzer High-sensitivity DNA chip. Libraries were sequenced on Illumina MiSeq platform (50 cycles for HNRNPK and 150 cycles for PTBP1, single-read). The details of all the linkers, primers, and adapters used in the study is described in Supplementary Table S3.

After MiSeq sequencing, reads were adapter trimmed using cutadapt (19) (version 2.7) and reads less than 18 bp in length were discarded (see Supplementary Table S3 for adapter sequences used). The libraries were then demultiplexed using an in-house program (splitByBarcode) based on a 6-mer sample barcode, and this barcode was stripped using fastx_trimmer (http://hannonlab. cshl.edu/fastx_ toolkit/) (20) (FASTX Toolkit version 0.0.14). Mapping was then performed against custom genome annotations with STAR (21) (version 2.5.0a). Each file was mapped to an annotation consisting of the full human genome (hg38) plus an additional sequence for the SINEUP-GFP construct. To retain multiple mapping reads we opted to keep all primary alignments regardless of how many secondary alignments could be found for the same read, rather than keeping only the uniquely mapping reads. The mapped reads for the two replicates of the HNRNPK seCLIP and input samples were merged at this stage using the bamtools (22) merge function (bamtools version 2.4.1).

SINEUP-GFP deletion mutants lacking PTBP1 or HNRNPK binding regions were synthesized by the commercial preparation service at GENEWIZ (Saitama, Japan). HEK293T/17 cells were prepared as described above (see cell culture and plasmid transfection), and EGFP up-regulation was measured by Western blotting assay (see Measuring protein up-regulation by Western blotting assay).

### GO enrichment analysis

The RNA-seq data (23) discussed in this section have been obtained from the Sequence Read Archive (SRA) and are accessible through the SRA accession number SRP111756 (https://www.ncbi.nlm.nih.gov/sra/?term=SRP111756). Raw FASTQ files for MCF7 (paired end) were downloaded and processed to remove rRNA reads, check overall quality and remove any orphan reads from the paired end samples. Resultant files were mapped to the human genome (hg38) using STAR (21). Gene counts were generated using htseq-count (24), after prior filtering of secondary mapped and unmapped alignments. Count files were used in DESeq2 (25) to generate a list of differentially expressed genes between control and siRNA knockdown for each cell fraction (cytoplasm, nucleus, whole cell extract). Genes with an FDR adjusted *P*-value of <0.05 were used for GO term enrichment analysis using the Bioconductor package topGO (https://bioconductor.org/packages/release/bioc/html/topGO.html) (26). The background for each analysis was the full list of genes with non-zero expression in that fraction. All GO terms had to have at least 10 annotated genes to be considered, and enrichment was tested using the Kolmogorov-Smirnov elim method, with a score of <0.05 considered significant.

### Statistical analysis

Statistical differences were measured using a paired, two-sided Student's *t-*test. Bar graphs were described as mean ± standard deviation (SD) from at least three independent experiments. Statistically significant changes relative to a negative control were represented with *P < 0.05, **P < 0.01. To test normality of all data sets, Kolmogorov–Smirnov test was used.

## RESULTS

### SINEUP-GFP enhances *EGFP* mRNA translation

**Figure 1.**
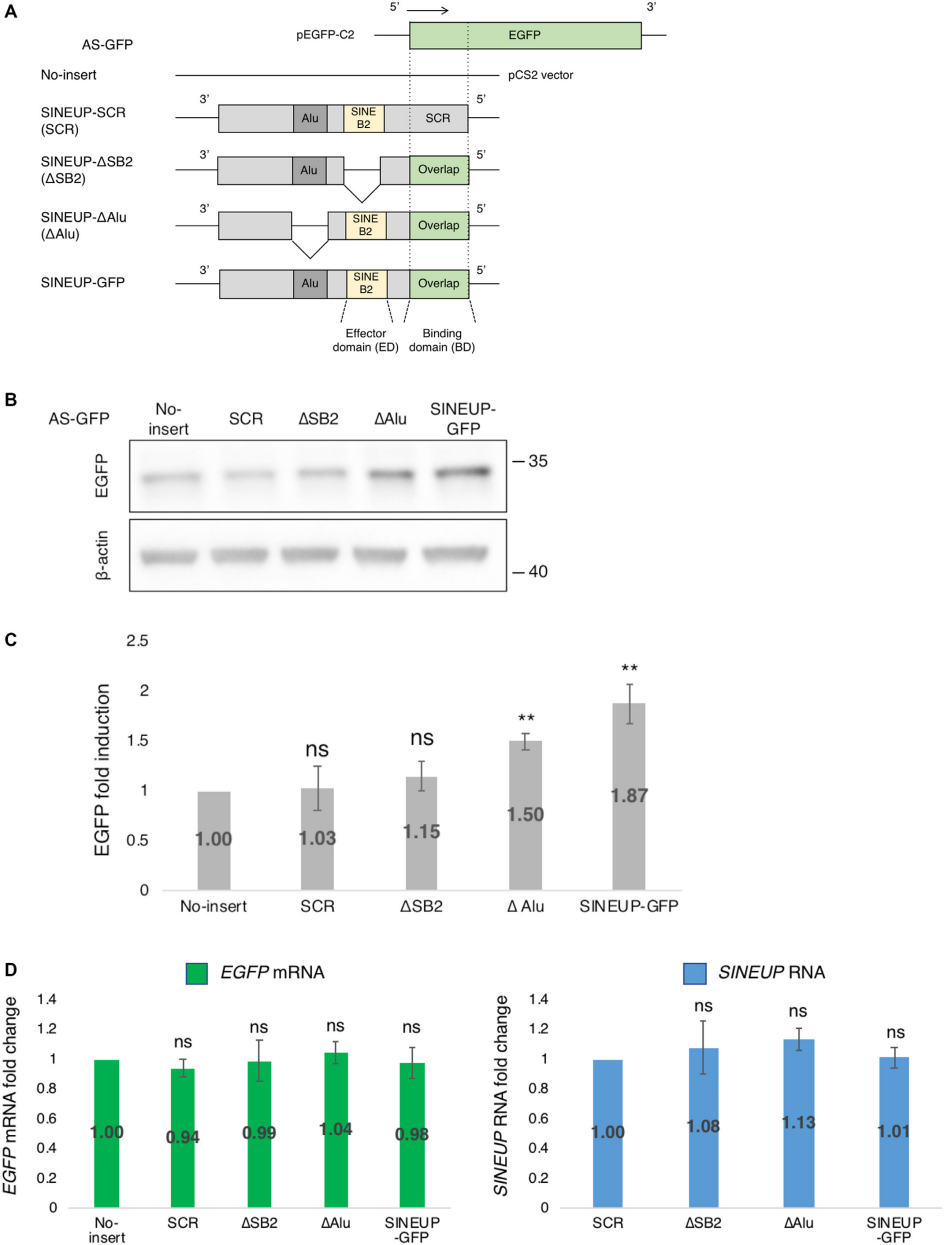
Enhancement of EGFP level by synthetic SINEUP-GFP. (**A**) Schematic representation of the SINEUP constructs used in this study. SINEUP-GFP contains the overlapping region with *EGFP* (binding domain, BD) and SINEB2 element (effector domain, ED). Domain deletion mutants constructed from SINEUP-GFP are shown: SINEUP-SCR (SCR) contains a scrambled sequence instead of the *EGFP* BD; SINEUP-delta SB2 (ΔSB2) has a deleted SINEB2 element; and SINEUP-delta Alu (ΔAlu) has a deleted Alu repeat element. (**B**) Translational up-regulation of EGFP by co-transfection of EGFP and SINEUP expression vectors. Western blotting image showing the effect of SINEUPs on the EGFP level; the result shown is representative of at least three independent experiments. (**C**) Quantification of the up-regulation of EGFP by co-transfection with EGFP and SINEUP vectors. **P < 0.01, ns: not significant, by Student's *t*-test. Data are means ± SD from at least three independent experiments. (**D**) Quantification of the *EGFP* mRNA and *SINEUP* RNA levels following co-transfection with EGFP and SINEUP expression vectors. Data are means ± SD from at least three independent experiments.

To confirm the translational up-regulation activity of SINEUP constructs, we produced synthetic SINEUPs targeting *EGFP* mRNA (Figure 1A). We previously reported that *SINEUP-GFP* enhances EGFP levels more efficiently when cloned into a pCS2+ plasmid (12) than when cloned into a pcDNA3.1 plasmid (9,10). The expression levels of RNA produced by different plasmids often differ due to different promoters, stability, and polyadenylation status, and in the current study we found that the level of *SINEUP* RNA transcripts (measured as copy number per cell) was ∼1.5-fold higher for pCS2+ than for pcDNA3.1 (see Supplementary Figure S1B for measurements details). Therefore, pCS2+ was used for all subsequent experiments. We then examined the EGFP up-regulation activities of SINEUP-GFP and a series of deletion mutants in HEK293T/17 cells; a BD mutant, with a scramble BD sequence (SINEUP-SCR); an ED deletion mutant (SINEUP-ΔSB2), and an Alu element deletion mutant (SINEUP-ΔAlu) (Figure 1A). Consistent with previous studies, synthetic SINEUP-GFP in pCS2+ showed ∼2-fold induction of EGFP levels compared to the no-insert control (vector containing no SINEUP construct) (Figure 1B, C). SINEUP-SCR and SINEUP-ΔSB2 did not significantly elevate the EGFP levels, but the Alu element deletion mutant (SINEUP-ΔAlu) and SINEUP-GFP enhanced EGFP levels, as expected. Because none of the constructs significantly affected the *EGFP* mRNA (Figure 1D), the results indicate that translation of *EGFP* mRNA was induced by SINEUP-GFP and SINEUP-ΔAlu, but not by SINEUP-SCR or SINEUP-ΔSB2.

### *SINEUP* RNAs co-localized with *EGFP* mRNAs in the cytoplasm

**Figure 2.**
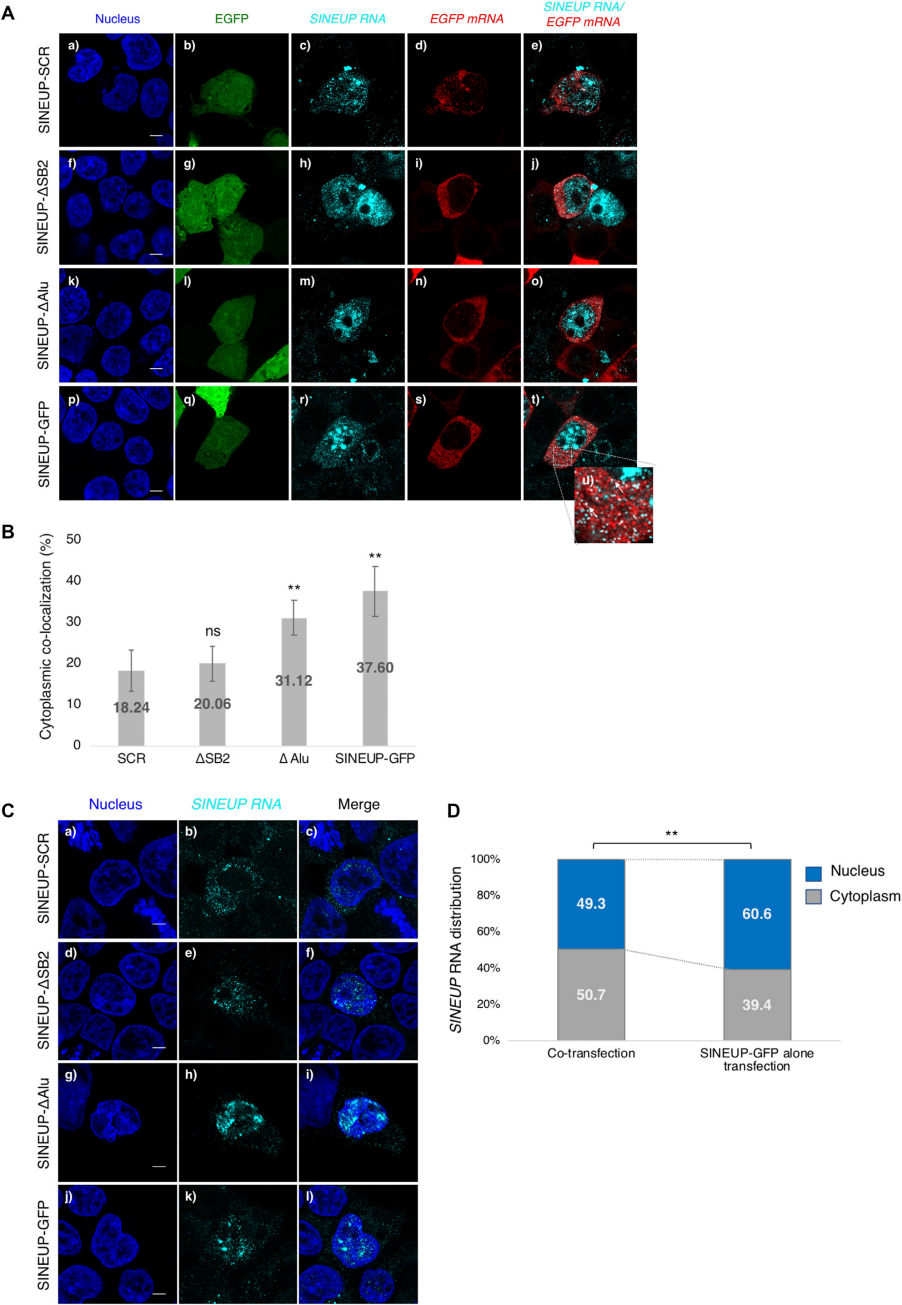
Co-localization of *SINEUP-GFP* RNAs with *EGFP* mRNAs in the cytoplasm. (**A**) Subcellular localization of *SINEUP* RNAs and *EGFP* mRNAs. Bars indicate 5 μm. (**B**) Comparison of the percentage co-localization of *EGFP* mRNAs and *SINEUP* RNAs in the cytoplasm. Data are means ± SD of at least 20 independent cell images. **P < 0.01, ns: not significant by Student's *t*-test. (**C**) Subcellular distribution of *SINEUP* RNAs following transfection with SINEUP expression vectors alone. Bars indicate 5 μm. (**D**) Quantitative comparison of the distribution of *SINEUP-GFP* RNA in the presence and absence of *EGFP* mRNA. The ratios of detected spots in the nucleus and cytoplasm were compared between co-transfection of SINEUP-GFP and EGFP vectors, and transfection of SINEUP-GFP vector alone. Data were collected from at least 10 independent cell images. **P < 0.01 by Student's *t*-test.

A previous study showed that the natural *SINEUP* RNA (AS-*Uchl1*) is transported to the cytoplasm upon rapamycin treatment, enhancing *Uchl1* mRNA translation (9). We hypothesized that the subcellular distribution of *SINEUP* RNAs has a key role in regulating target mRNA translation. To elucidate the kinetic distribution of *EGFP* mRNA and *SINEUP* RNA, we performed RNA FISH (fluorescence *in situ* hybridization) following co-transfection of EGFP and SINEUP expression vectors (SINEUP-GFP or the deletion mutants) into HEK293T/17 cells. We observed that *EGFP* mRNAs were predominantly localized in the cytoplasm (Figure 2A, d, i, n, s), whereas the *SINEUP* RNAs were distributed both in the nucleus and the cytoplasm (Figure 2A, c, h, m, r). In the nucleus, *SINEUP* RNAs were located throughout the nucleoplasm, but not in the nucleolus.

*SINEUP* RNAs formed intensively clustered spots, which were partially co-localized with several nuclear organelle markers (Supplementary Figures S2A and S2B for cytoplasmic organelle markers). In the cytoplasm, *SINEUP* RNAs co-localized with *EGFP* mRNAs, appearing as numerous small dots distributed throughout the cytoplasm (Figure 2A, u, arrows). Co-localization of *EGFP* mRNA and *SINEUP* RNA in the cytoplasm was observed more frequently for SINEUP-GFP (37.60%) and SINEUP-ΔAlu RNAs (31.12%) than for *SINEUP* RNAs with impaired translational up-regulation activity (i.e. SINEUP-SCR and SINEUP-ΔSB2) (Figure 2B); SINEUP-ΔAlu and SINEUP-GFP showed more frequent overlapping peaks (arrows) compared with SINEUP-SCR and SINEUP-ΔSB2 (Supplementary Figure S3A). This indicated that the BD and ED domains contribute both to the up-regulation of translation, and to the co-localization of *EGFP* mRNAs and *SINEUP* RNAs. When the EGFP expression vector was transfected alone, most *EGFP* mRNAs were distributed in the cytoplasm (Supplementary Figure S3B), as was observed for co-transfection (Figure 2A, d, i, n, s). A similar cytoplasmic pattern was observed for *EGFP* mRNA when co-transfected with all SINEUP mutants (Supplementary Figure S3C). In contrast, when the expression vectors for *SINEUP* RNAs were transfected alone, *SINEUP* RNAs were preferentially distributed in the nucleus (Figure 2C, e, h, k). To compare the subcellular distribution of *SINEUP* RNAs between the cells co-transfected with EGFP and SINEUP vectors to those transfected with SINEUP alone, the signals were detected in both the whole cell and the nuclear region alone using icy Spot Detector (Supplementary Figure S3D). The percentage of *SINEUP-GFP* RNA detected in the nucleus was 60.6% when the SINEUP-GFP vector was transfected alone (Figure 2D); this was significantly reduced to 49.3% when the SINEUP vector was co-transfected with the EGFP vector, meaning more *SINEUP-GFP* RNA was shifted to the cytoplasm. A similar finding was observed for SINEUP-ΔAlu, but no significant differences were observed for SINEUP-SCR or SINEUP-ΔSB2 (Supplementary Figure S4A–C). As supported by qPCR measurements of RNA expression, subcellular distribution of *SINEUP-ΔAlu* and *SINEUP-GFP* RNA shifted to the cytoplasm when those transcripts were co-transfected with *EGFP* mRNA, while cytoplasmic *SINEUP* RNAs were reduced when the SINEUP vector was transfected alone (except for SINEUP-SCR) as compared to SINEUP vector with EGFP vector co-transfection (Supplementary Figure S4D). Consistent with these observations, translational up-regulation was enhanced by exporting the *SINEUP* RNAs into the cytoplasm in the presence of *EGFP* mRNA.

### Identification and functional analysis of *SINEUP* RNA binding proteins

**Figure 3.**
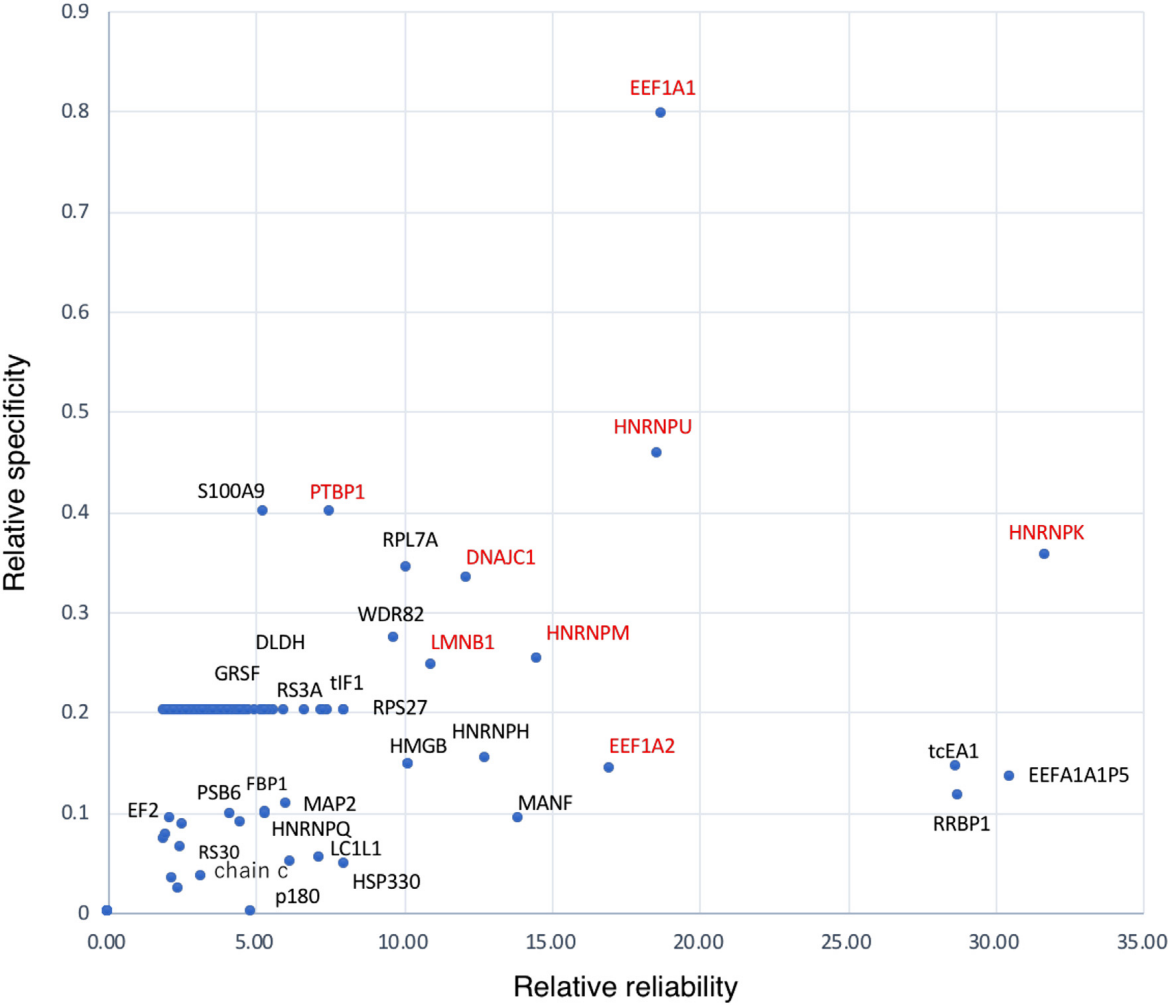
SINEUP-GFP RNA binding proteins. Detected RNA binding proteins (RBPs) were plotted with a reliability of RBPs on the x-axis that was calculated by the average of the SINEUP-GFP *MASCOT* score; reliability = sum of the MASCOT score/ n, and with a specificity of RBPs in target samples on the y axis that was the sum of the MASCOT score divided by the total MASCOT score of other mutants; specificity = (sum of the MASCOT score/sum of total other mutants MASCOT score)/*n*. *n* ≧ 3. Proteins that were detected by beads and LacZ probes were omitted. The proteins selected knocked down for the following siRNA mediated experiments are indicated in red.

**Figure 4.**
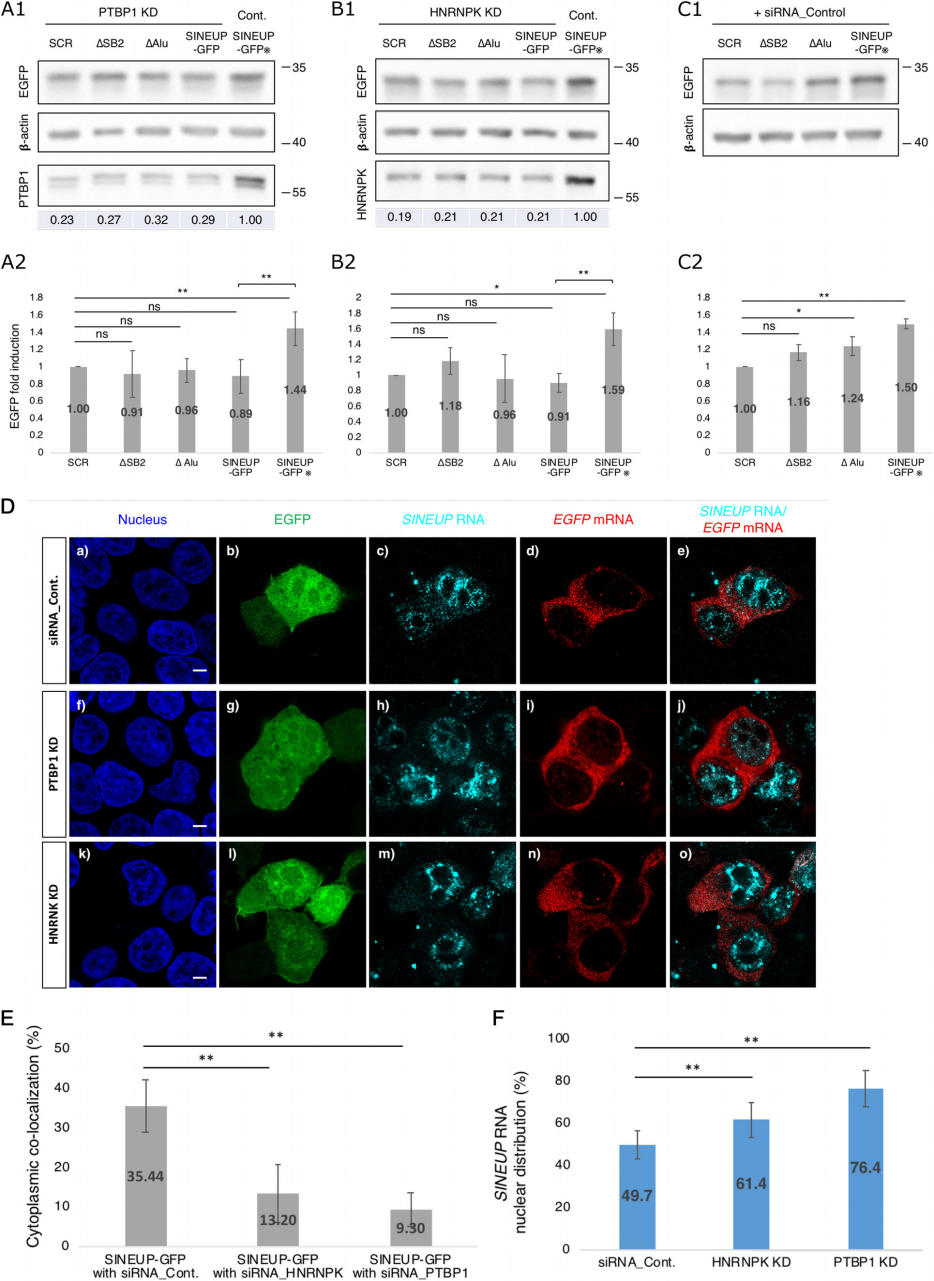
Knockdown of SINEUP RBPs. (**A1, B1**) Representative Western blotting images of knockdown (KD) of PTBP1 (**A1**) and HNRNPK (**B1**) mediated by siRNA-PTBP1 and siRNA-HNRNPK, respectively. Numbers under the bottom row indicate knockdown efficiency compared with cells co-transfected with SINEUP-GFP and negative control siRNA (SINEUP-GFP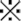 in **C1** and **C2**). (**C1**) Representative Western blotting images of transfection with negative control siRNA. (**A2, B2, C2**) Protein levels of EGFP were quantified by Western blotting analysis. EGFP expression levels were normalized to that of β-actin. Fold induction of EGFP was calculated relative to cells transfected with the siRNA indicated in the above panels A1, B1, or C1 respectively. **P < 0.01, *P < 0.05; ns: not significant by Student's *t*-test. Data are means ± SD of at least three independent experiments. (**D**) Representative FISH images following knockdown (KD) of PTBP1 (f-j) or HNRNPK (k-o) by siRNAs, and negative control siRNA; siRNA_Cont. (a–e). Bars indicate 5 μm. (**E**) Quantitative comparison of co-localization of *EGFP* mRNAs and *SINEUP* RNAs in the cytoplasm when PTBP1 (D, j) or HNRNPK (D, o) were knocked down. **P < 0.01 by Student's *t*-test. Data are means ± SD of 10 individual cell images. (**F**) Quantitative nuclear distribution of *SINEUP-GFP* RNAs following knockdown of PTBP1 (D, h) or HNRNPK (D, m) by siRNAs; the results are compared with the cells transfected negative control siRNA; siRNA_Cont (D, c). For both PTBP1 and HNRNPK, the ratio of *SINEUP-GFP* RNA levels in the nucleus and the cytoplasm were compared between the knockdowns and the negative control. **P < 0.01 by Student's *t*-test. Data are means ± SD of at least 10 independent cell images.

We hypothesized that *SINEUP* RNA binding proteins (RBPs) may play a crucial role in EGFP expression. To identify SINEUP RBPs in the cells, we used a modified version of the Chromatin Isolation by RNA Purification (ChIRP) method (15) (Supplementary Figures S5A, B) followed by mass spectrometry (MS) analysis. By carrying out three or more independent experiments on SINEUP-SCR, SINEUP-ΔSB2, SINEUP-ΔAlu and SINEUP-GFP transfection, we detected several SINEUP RBPs. To determine which RBPs are the most important for the translational up-regulation activity of SINEUP-GFP, we selected several candidate SINEUP-GFP RBPs with high reliability and specificity by the calculation of score from mass spectrometry database searched engine Mascot (see method, Figure 3; Supplementary Table S1) while non-specific bound proteins, which were also detected in the samples with beads and labelled as LacZ probe, were removed to identify specific RBPs (Supplementary Figure S5B). We then compared the SINEUP-GFP RBPs with those for the other SINEUP mutants (Supplementary Figure S5C–E; Supplementary Table S1) and observed that several nucleocytoplasmic shuttling-related proteins were specifically enriched as SINEUP-GFP RBPs (Figure 3). After excluding ribosomal proteins, we performed siRNA-mediated knockdown of enriched proteins (PTBP1, HNRNPK, DNAJC1, EEF2, EF1A1, HNRNPM, HNRNPU and LMNB1) in the scatterplot (in red at Figure 3) to assess their effects on the up-regulation of SINEUP-GFP protein translation (Figure 4; Supplementary Figure S6A–F). The experiments revealed that knockdown of either PTBP1 (Figure 4A1 and A2, and Supplementary Figure S7A) or HNRNPK (Figure 4B1 and B2, and Supplementary Figure S7A) significantly decreased the translational up-regulation activity of SINEUP-GFP. On the other hand, transfecting cells with scramble siRNA (negative control) did not affect the translational up-regulation activity of SINEUP-GFP (Figure 4C1 and C2, SINEUP-GFP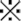, and Supplementary Figure S7B). Knockdown of EF1A1 decreased the up-regulation of translation for EGFP, but also affected β-actin levels as a non-specific effect (Supplementary Figure S6C), suggesting it affected global translational pathways. Interestingly, knockdown of PTBP1 (Figure 4D, f–j) and HNRNPK (Figure 4D, k–o) significantly reduced the co-localization of *EGFP* mRNA and *SINEUP-GFP* RNA in the cytoplasm compared with the cells transfected by negative control siRNA; siRNA_Cont. (Figure 4D, a-e) (Figure 4E). Furthermore, it significantly increased *SINEUP-GFP* RNA nuclear retention: 76.4% for PTBP1 knockdown and 61.4% for HNRNPK knockdown versus 49.7% in control cells (Figure 4F), suggesting a decrease in cytoplasmic SINEUP RNAs. In particular, knocking down PTBP1 significantly reduced cytoplasmic *SINEUP* RNAs in the cytoplasm (Supplementary Figure S8A), while knockdown of HNRNPK reduced both *SINEUP* RNAs and *EGFP* mRNAs across the whole cell fraction (Supplementary Figure S8A and B). These results suggest that *SINEUP* RNAs and *EGFP* mRNAs were not sufficient for SINEUP to function when HNRNPK levels are reduced after knockdown. The *SINEUP* RNA level in the whole cell lysate (WCL) following PTBP1 knockdown was not significantly changed, with reduction of *SINEUP* RNA only seen at the cytoplasmic level following PTBP1 knockdown. This suggests that PTBP1 does not have an effect on the transcript expression level and *EGFP* mRNA translation machineries, but instead has an effect on *SINEUP* RNA subcellular localization, consistent with changes in subcellular localization following PTBP1 overexpression (see Supplementary Figure S9).

**Figure 5.**
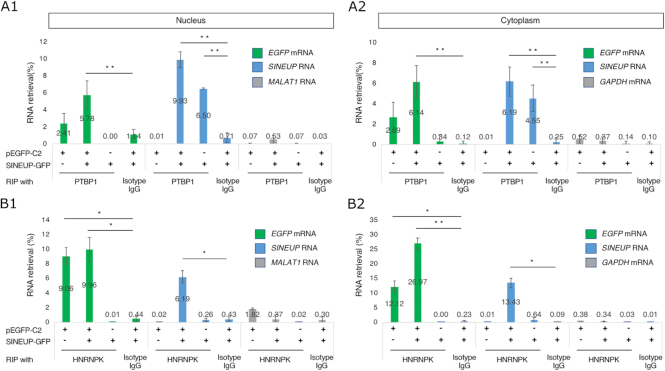
RNA immunoprecipitation. (**A1**, **A2**) RNA immunoprecipitation (RIP) with PTBP1 antibody in the nucleus (**A1**) and cytoplasm (**A2**). Isotype IgG was used as the negative antibody control. Transcripts from two of the most highly expressed housekeeping genes, *MALAT1* and *GAPDH*, were used as negative controls in the nucleus and cytoplasm, respectively. *P < 0.05, **P < 0.01 by Student's *t*-test. Data are means ± SD of at least three independent experiments. (**B1**, **B2**) RNA immunoprecipitation (RIP) with HNRNPK antibody or isotype immunoglobulin G (Isotype IgG; negative control) in the nucleus (**B1**) and cytoplasm (**B2**). Cell lysates co-transfected with pEGFP-C2 and SINEUP-GFP vectors or transfected with either vector alone were tested.

In the knockdown of HNRNPK, we observed that the reduction of *EGFP* mRNA and *SINEUP* RNA co-localization resulted in the loss of EGFP enhancement. Since HNRNPK is a highly expressed protein related to several biological processes (27–29), and associates with several target transcripts to contribute to their subcellular localization (23), we investigated whether our knockdown experiment has an effect on global translation. To do this we used published RNA-seq data from MCF7 cells with a knockdown timing similar to that employed in our experiment (23). As results, analysis of gene ontology (GO) terms after knockdown of HNRNPK in MCF7 cells suggested that translational regulation is not affected by perturbation of HNRNPK, as the number of genes involved in ‘translation’ is negligible, while the cellular response seems to involve other processes (see Supplementary Figure S10 and Materials and Methods section ‘GO enrichment analysis’). As a caveat, although the experiment was performed in MCF7 cells, which may somehow differ from the HEK293T cells, translation is generally a conserved cellular function. Therefore, PTBP1 and HNRNPK may mainly participate in the nucleocytoplasmic shuttling of *SINEUP* RNAs. To better understand the role of PTBP1 and HNRNPK interactions in *SINEUP-GFP* RNA nucleocytoplasmic shuttling, we conducted an RNA immunoprecipitation (RIP) assay of RNA–protein interactions with PTBP1 and HNRNPK proteins in the nucleus and cytoplasm. *SINEUP-GFP* RNAs were pulled down with PTBP1 both in the nucleus (Figure 5A1) and cytoplasm (Figure 5A2) and *EGFP* mRNAs were pulled down with HNRNPK both in the nucleus (Figure 5B1) and cytoplasm (Figure 5B2). These observations show that (a) PTBP1 protein was able to bind to *SINEUP-GFP* RNA or the *EGFP-SINEUP* RNA complex in either the nucleus or the cytoplasm, but not to *EGFP* mRNA alone; and (b) HNRNPK protein was able to bind to *EGFP* mRNA or the *EGFP–SINEUP* RNA complex in either the nucleus or the cytoplasm, but did not bind to *SINEUP-GFP* RNA alone. Taken together, these findings indicate that PTBP1 and HNRNPK play a role in the formation of the RNA-protein complexes and participate in the kinetic distribution of these RNA–protein complexes.

### SINEUP RBPs drive subcellular localization of *SINEUP* RNAs and participate in translational initiation assembly

**Figure 6.**
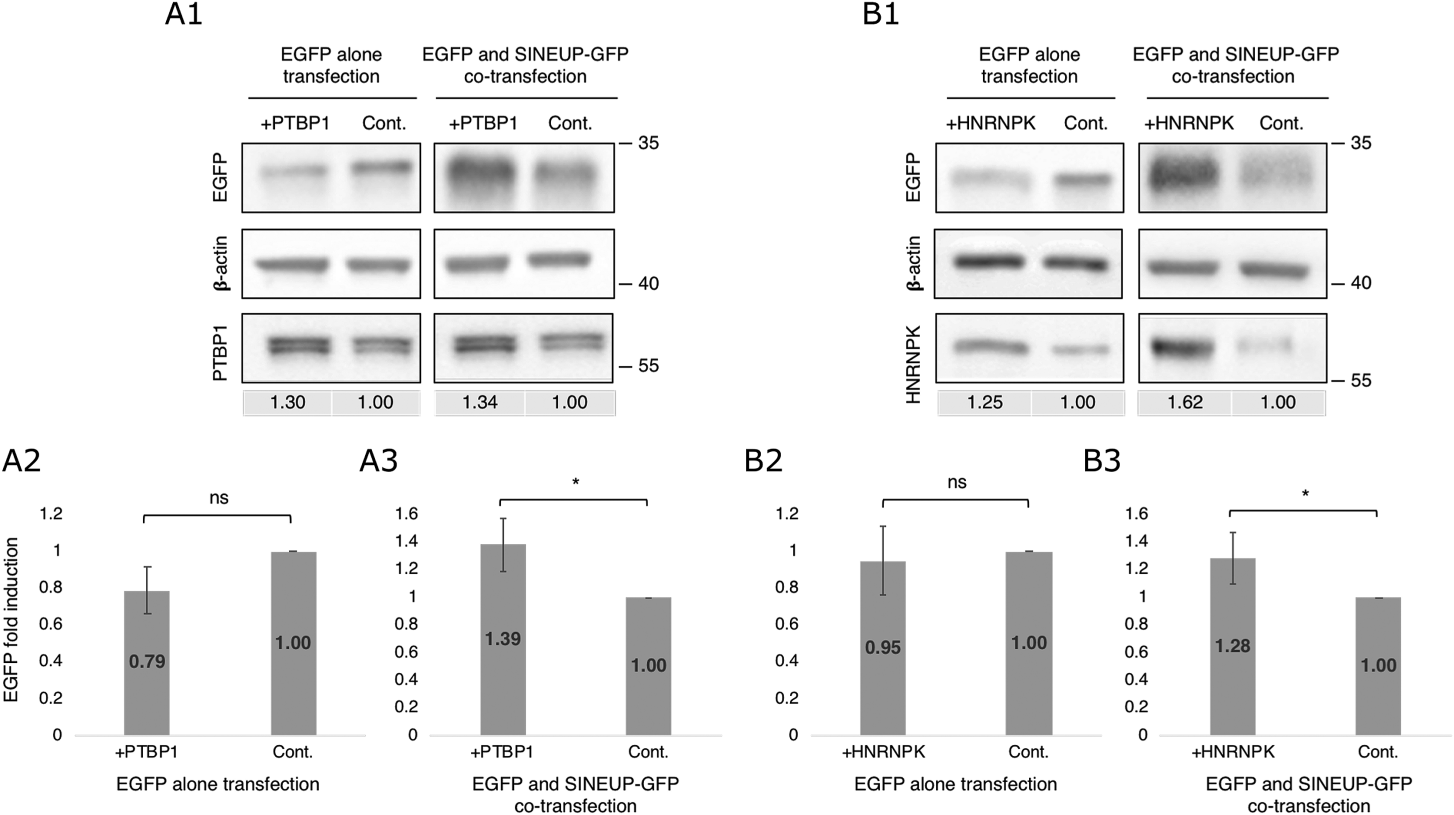
Overexpression of SINEUP RBPs. (**A1**, **B1**) Representative Western blotting images comparing EGFP expression after overexpression of PTBP1 (+PTBP1) (**A1**) or HNRNPK (+HNRNPK) (**B1**) with that in the non-overexpressing control (Cont.). Numbers under the image show the overexpression efficiency compared with controls. (**A2**, **A3**, **B2**, **B3**) Quantification of EGFP levels after non-/overexpression of PTBP1 (**A2**, **A3**) or HNRNPK (**B2**, **B3**) when cells were transfected with EGFP vector alone (**A2**, **B2**) or co-transfected with EGFP and SINEUP-GFP vectors (**A3**, **B3**). *P < 0.05, ns: not significant by Student's *t*-test. Data are means ± SD of at least three independent experiments.

We next wondered whether EGFP levels would be further enhanced by overexpression of PTBP1 and HNRNPK proteins. To address this question, we transfected cells with either PTBP1 or HNRNPK expression vector. The EGFP level was moderately, but significantly increased by overexpression of PTBP1 (Figure 6A1 and A3) or HNRNPK (Figure 6B1 and B3) in the cells co-transfected with EGFP and SINEUP-GFP vectors, but not in those transfected with EGFP vector alone (Figure 6A2 and B2). This finding suggests that PTBP1 and HNRNPK formed a protein–*SINEUP* RNA complex and functionally enhanced EGFP levels.

**Figure 7.**
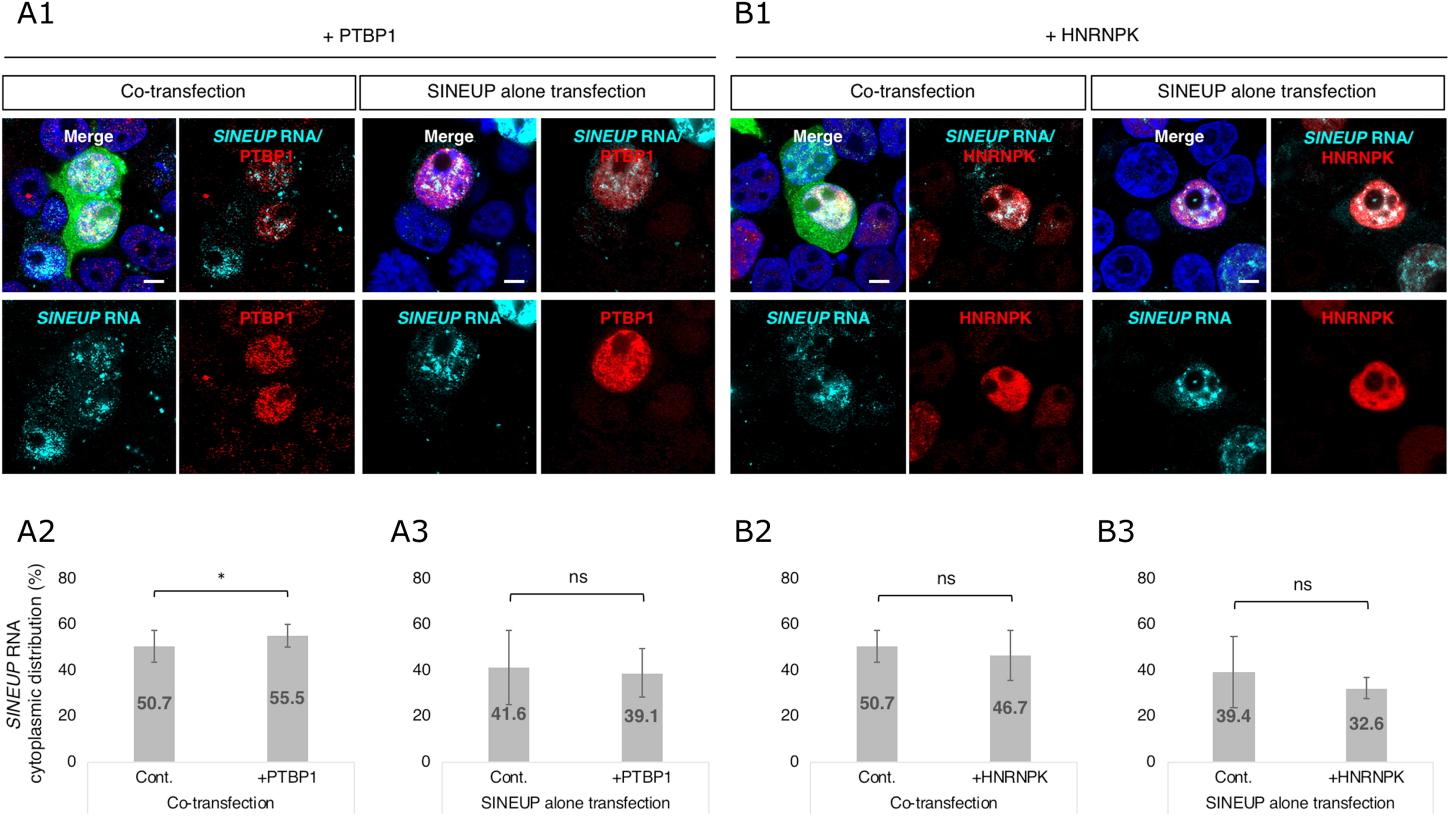
Subcellular distribution of *SINEUP* RNAs after overexpression of SINEUP RBPs. (**A1**, **B1**) Representative RNA FISH with immunofluorescence images of the subcellular distribution of *SINEUP* RNAs and SINEUP RBPs in cells overexpressing PTBP1 (+PTBP1) (**A1**) or HNRNPK (+HNRNPK) (**B1**). Images for cells co-transfected with EGFP and SINEUP-GFP vectors (left images, **A1** and **B1**) were compared with cells transfected with SINEUP-GFP vector alone (right images, **A1** and **B1**). Bars indicate 5 μm. (**A2**, **A3**, **B2**, **B3**) Quantitative comparison of *SINEUP-GFP* RNA distribution between cells overexpressing PTBP1 (**A2**, **A3**) or HNRNPK (**B2**, **B3**) and cells without overexpression (Cont.). Results for cells co-transfected with EGFP and SINEUP-GFP vectors (**A2** and **B2**) and those transfected with SINEUP-GFP vector alone (**A3**, **B3**) are shown. *SINEUP* RNA signals were detected using Icy Spot Detector. The ratio of spots in the nucleus and the cytoplasm were compared between overexpression and non-overexpression of SINEUP RBPs. *P < 0.05 by Student's *t*-test. Data are means ± SD of at least 10 independent cell images.

To evaluate the effect of SINEUP RBPs on the subcellular distribution of *SINEUP* RNA, we overexpressed PTBP1 (Figure 7A) or HNRNPK (Figure 7B) in the cells and then co-transfected the cells with EGFP and SINEUP-GFP vectors, or with SINEUP-GFP vector alone. Some nuclear *SINEUP-GFP* RNAs were preferentially shuttled into the cytoplasm when *EGFP* and *SINEUP-GFP* transcripts were co-transfected into cells overexpressing PTBP1 compared to cells with normal PTBP1 levels (Figure 7A2: Supplementary Figure S9A); this difference was not seen when the cells were transfected with SINEUP-GFP vector alone (Figure 7A3). Induction of PTBP1 did not directly drive *SINEUP-GFP* RNA from the nucleus to the cytoplasm without the presence of *EGFP* mRNAs. In contrast to PTBP1, overexpression of HNRNPK had no significant effect on the subcellular distribution of *SINEUP-GFP* RNAs (Figure 7B2 and B3; Supplementary Figure S9A). Induction of PTBP1 or HNRNPK did not affect *EGFP* mRNA sub-cellular distribution (Supplementary Figure S9B). Taking these results together, these findings indicate that PTBP1 and HNRNPK participate in nucleocytoplasmic shuttling of RNA-protein complexes and further act to regulate translation after shuttling into the cytoplasm.

**Figure 8.**
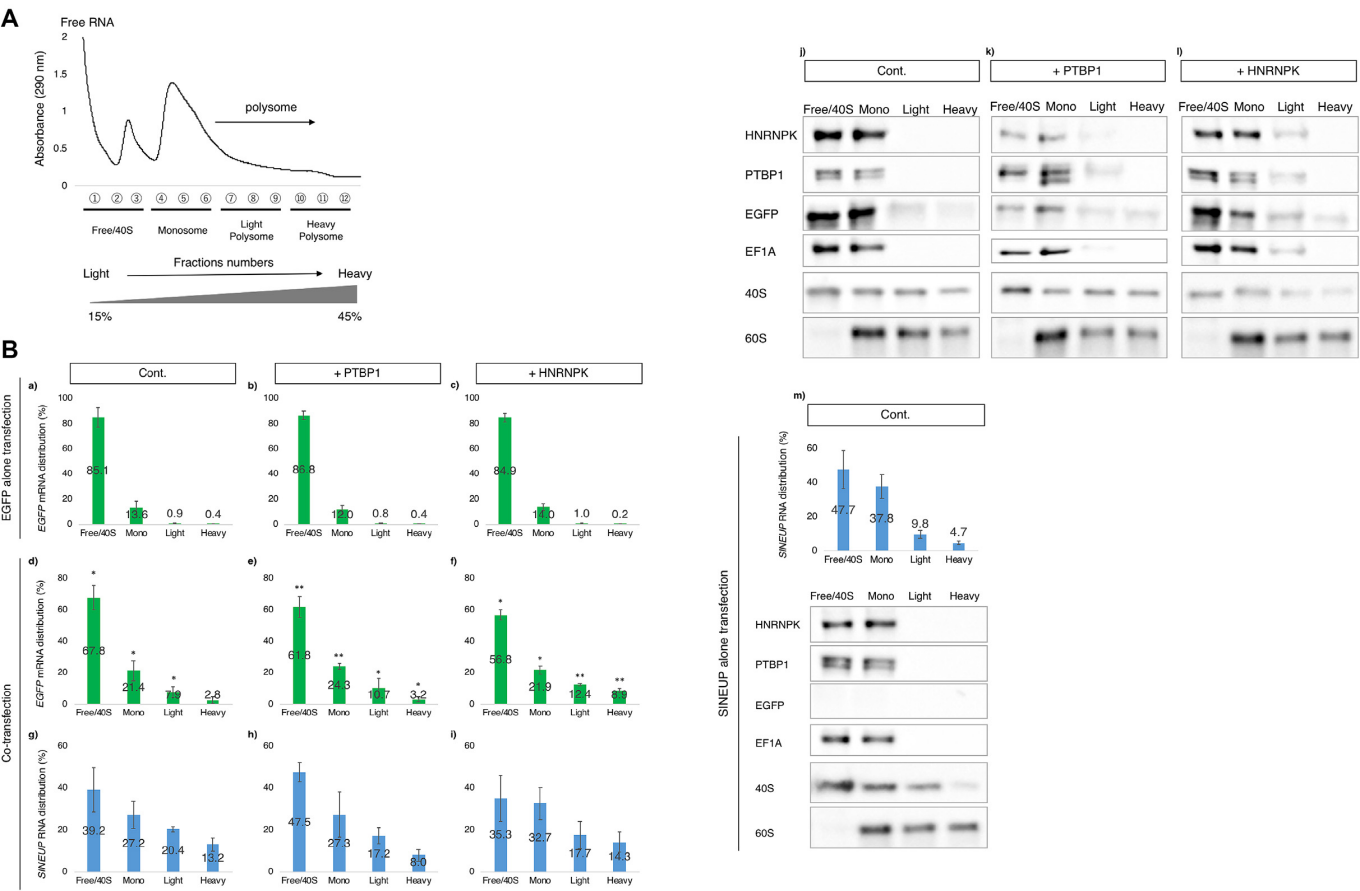
RNA distribution in polysome fractions obtained from cells overexpressing SINEUP RBPs. (**A**) Representative polysome gradient profile with optical density (290 nm). Fractions 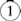 and 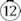 correspond to the 15% and 45% sucrose fractions, respectively. (**B**) Polysome profiling of *EGFP* mRNA (green) and *SINEUP-GFP* RNA (blue). RNA distribution was quantified by RT-qPCR. Each fraction of *EGFP* mRNA in cells co-transfected with EGFP and SINEUP-GFP vectors (d–f) was compared with the corresponding fraction in cells transfected with EGFP vector alone (a–c). *P < 0.05, **P < 0.01 by Student's *t*-test. Data are means ± SD from three independent experiments. Fractions of *SINEUP* RNA in cells co-transfected with EGFP and SINEUP-GFP vectors are shown in g-i, and those in cells transfected with *SINEUP* RNA alone are shown in m. The protein distribution in polysome fractions with non-/overexpressed PTBP1 or HNRNPK were tested (j-m). Equal volumes of solution were pooled as Free/40S (Free or 40S binding RNA fraction from 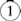
–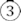 in A), Mono (monosome fraction from 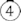
–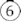), Light (light polysome fraction from 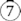
–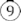), and Heavy (Heavy polysome fraction from 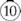
–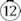) and were applied to 10% SDS gels to detect each target protein. Western blotting images representative of least three independent experiments are shown.

To gain further insights into the molecular mechanism of translational enhancement, we analyzed the distributions of the RNAs and RBPs in polysome fractions of cells overexpressing PTBP1 and HNRNPK. The cytoplasmic lysate was separated into 12 fractions using a 15–45% sucrose gradient (Figure 8A). In co-transfected cells, *EGFP* mRNAs were slightly shifted into heavier polysomes in all cases (control, Figure 8B, d; PTBP1 overexpression, Figure 8B, e; HNRNPK overexpression, Figure 8B, f) compared with the corresponding cells when EGFP vector alone was transfected (control, Figure 8B, a; PTBP1 overexpression, Figure 8B, b; HNRNPK overexpression, Figure 8B, c). *SINEUP-GFP* RNA co-sedimented with *EGFP* mRNA in the heavy polysome fractions when *EGFP* mRNA was present (Figure 8B, g, h, i). Although we observed in the FISH analysis (Figure 2C and D) that most *SINEUP-GFP* RNA was retained in the nucleus when the cells were transfected with SINEUP-GFP alone, more than 85% of the cytoplasmic *SINEUP-GFP* RNA sedimented in the fractions containing Free/40S binding RNAs (47.7%) and monosomes (37.8%) (Figure 8B, m). This implies that the cytoplasmic *SINEUP-GFP* RNA may participate in an initial phase of translation. We conducted Western blotting analysis of the co-distribution of SINEUP RBPs and RNAs in the polysome fractions (Figure 8B, j, k, l). The analysis revealed that HNRNPK co-sedimented with the RNAs and PTBP1 in the light polysome fractions when HNRNPK was overexpressed (Figure 8B, l). PTBP1 also co-sedimented with the RNAs in the light polysome fractions when PTBP1 was overexpressed (Figure 8B, k). Additionally, PTBP1 co-sedimented with *SINEUP-GFP* RNA from the Free/40S fractions and monosome fractions even when SINEUP-GFP vectors were transfected alone (Figure 8B, m). This indicates that *SINEUP* RNAs associate with PTBP1 and may recruit ribosome subunits to contribute to the formation of translational initiation complexes, including elongation factor EF1A, to participate in the initial phases of translation.

### The SINEUP RBPs are crucial for enhancement of target mRNA translation and bind with several specific regions on SINEUP transcripts

**Figure 9.**
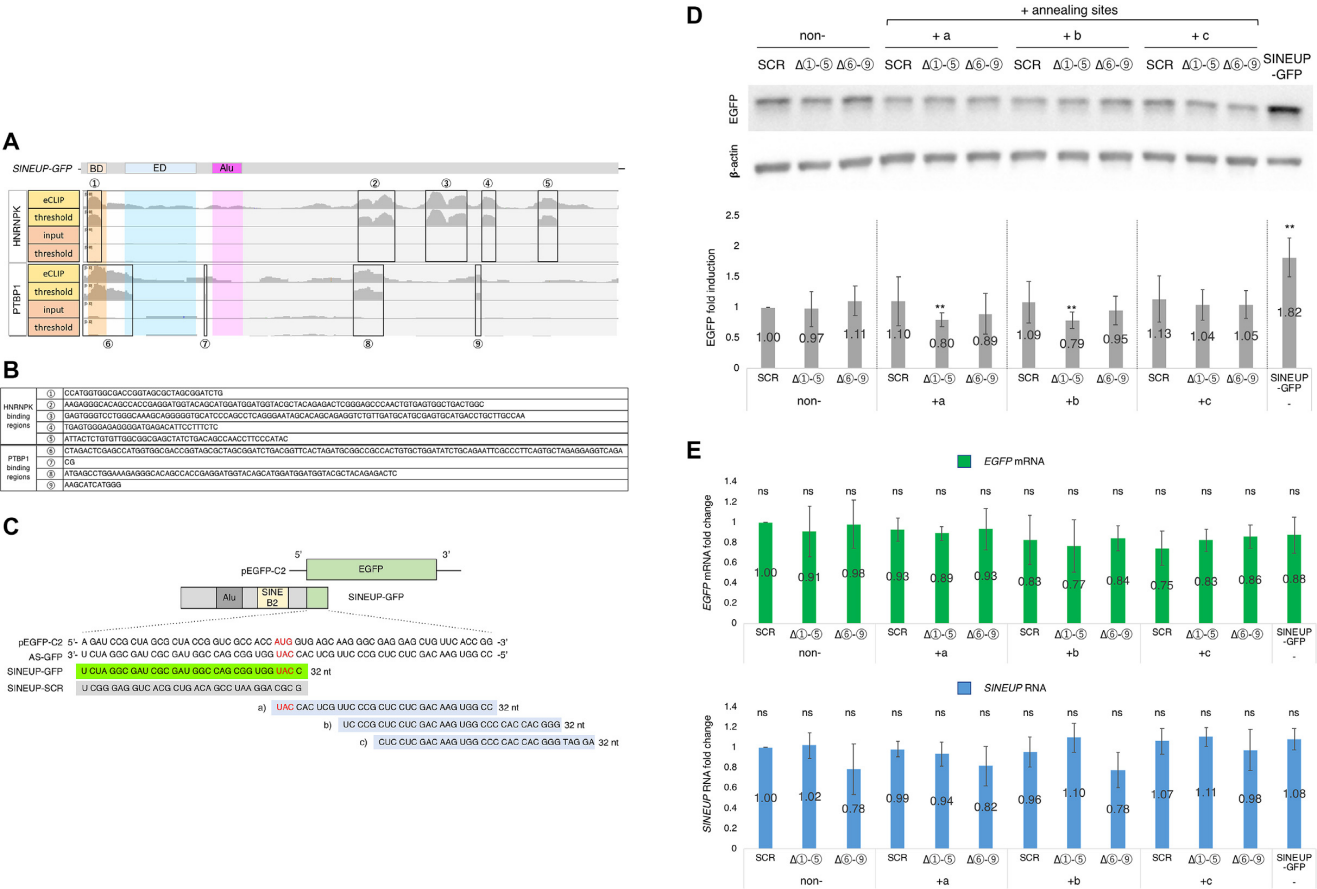
Identification of the SINEUP RBPs binding regions by seCLIP-seq analysis. (**A**) Read coverage along SINEUP-GFP shown by seCLIP with HNRNPK or PTBP1. Labeled boxes show the identified binding regions with HNRNPK (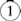
–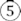) and PTBP1 (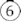
–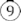) on *SINEUP-GFP* transcripts. (**B**) Sequences of binding sites of HNRNPK (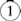
–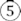) and PTBP1 (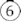
–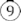) on *SINEUP-GFP* transcripts shown in A. (**C**) Schematic representation of annealing sites (a), (b) and (c) with the SINEUP-GFP and SCR mutant are shown. (**D**) Representative Western blotting image on the EGFP level (top) and quantification of the EGFP level (bottom). EGFP vector and the mutants were co-transfected in HEK-293T/17. **P < 0.01, ns: not significant, by Student's *t*-test. Data are means ± SD from at least three independent experiments. The SINEUP deletion mutants Δ
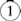
–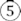 (deleted HNRNPK binding regions from SINEUP-GFP), and Δ
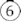
–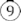 (deleted PTBP1 binding regions from SINEUP-GFP) were shown in A and B, and annealing sites are shown in C. (**E**) Quantification of *EGFP* mRNA and *SINEUP* RNA levels following co-transfection with EGFP and SINEUP expression vectors. Data are means ± SD from at least three independent experiments. The SINEUP deletion mutants Δ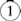-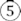 (deleted HNRNPK binding regions from SINEUP-GFP), and Δ
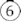
–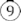 (deleted PTBP1 binding regions from SINEUP-GFP) were shown in A and B, and annealing sites were shown in C. ns: not significant by Student's *t*-test. Data are means ± SD from at least three independent experiments.

To determine the specific binding sites of PTBP1 and HNRNPK on *SINEUP-GFP* RNA, we performed seCLIP; single-end enhanced crosslinking and immunoprecipitation assay (Figure 9A and B). We then examined the ability of binding region deletion mutants (SINEUP-ΔHNRNPK binding regions; Δ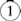-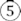, and SINEUP-ΔPTBP1 binding regions; Δ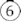-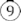, respectively) with different annealing sites (+a, +b, +c in Figure 9C) to up-regulate EGFP translation. We found that all the mutant, either lacking the sense-antisense region or those where the binding region was shifted outside the HNRNPK and PTBP1 binding regions (Δ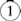-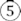, Δ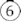-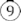), were ineffective to induce SINEUP activity (Figure 9D). The RNA level of *EGFP* mRNAs and *SINEUP* RNAs did not significantly change (Figure 9E), as expected. This shows that inhibition of PTBP1 and HNRNPK binding to *SINEUP-GFP* RNA at specific regions especially at surrounding BD results in the loss of EGFP up-regulation, therefore, the association of *SINEUP-GFP* RNA with PTBP1 and HNRNPK is crucial for its up-regulation activity.

### The SINEUP RBPs are important for up-regulation of endogenous target mRNA translation.

**Figure 10.**
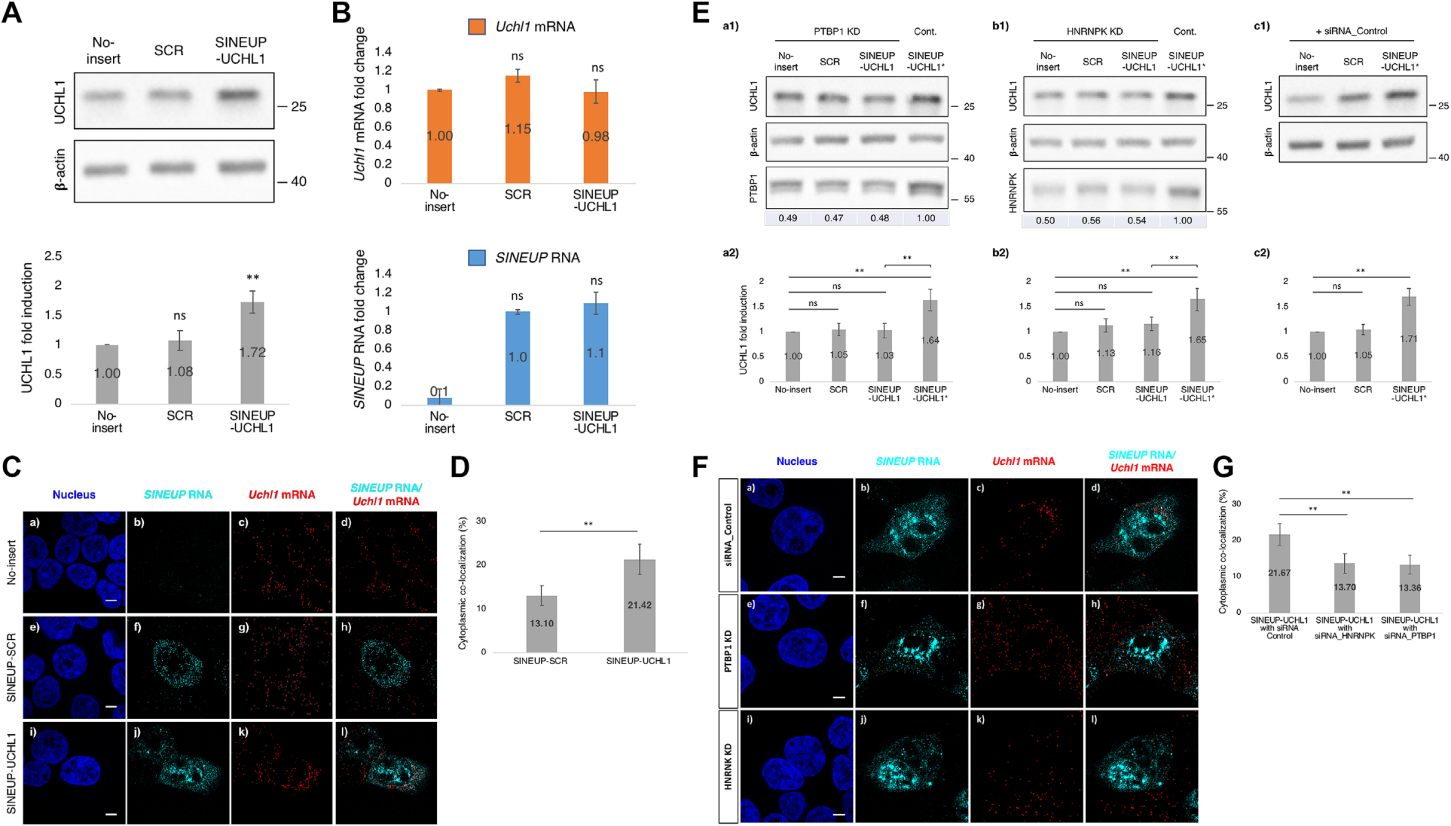
Enhancement of UCHL1 by synthetic SINEUP-UCHL1. (**A**) Translational up-regulation of UCHL1 by transfection of SINEUP-UCHL1 expression vectors. Representative Western blotting image on the UCHL1 protein level (top) and quantification of the UCHL1 level (bottom). **P < 0.01, ns: not significant, by Student's *t*-test. Data are means ± SD from at least 3 independent experiments. (**B**) Quantification of the *UCHL1* mRNA and *SINEUP* RNA levels following transfection with SINEUP expression vectors. Data are means ± SD from at least three independent experiments. (**C**) Subcellular localization of *SINEUP* RNAs and *Uchl1* mRNAs. Bars indicate 5 μm. (**D**) Quantitative comparison of co-localization of *Uchl1* mRNAs and *SINEUP* RNAs in the cytoplasm. **P < 0.01 by Student's *t*-test. Data are means ± SD of more than 10 individual cell images. (**E**) Translational up-regulation of UCHL1 by transfection with SINEUP-UCHL1 expression vectors. Representative Western blotting images of knockdown (KD) of PTBP1 and HNRNPK mediated by siRNA_PTBP1 (a1,2) and siRNA HNRNPK (b1,2), respectively. Numbers under the bottom row indicate knockdown efficiency compared with the cells transfected with SINEUP-UCHL1 and negative control siRNA (c1,2, SINEUP-UCHL1*). (**F**) Representative FISH images following knockdown (KD) of PTBP1 (e–h) or HNRNPK (i–l) by siRNAs. Bars indicate 5 μm. (**G**) Quantitative comparison of co-localization of *Uchl1* mRNAs and *SINEUP* RNAs in the cytoplasm when PTBP1 (F, h) or HNRNPK (F, l) were knocked down. **P < 0.01 by Student's *t*-test. Data are means ± SD of more than 10 individual cell images.

To examine whether PTBP1 and HNRNPK are important for enhancement of endogenous target translation, we designed SINEUP-UCHL1 and transfected into HEK293T/17 cells. Consistent with the GFP studies, SINEUP-UCHL1 showed ∼2-fold induction of UCHL1 protein levels compared with the No-insert control (vector without SINEUP) and SINEUP-SCR (Figure 10A). On the other hand, none of them significantly affect the *Uchl1* mRNA level (Figure 10B). In order to investigate the distribution of *Uchl1* mRNAs and *SINEUP* RNAs in the cells, we performed RNA FISH after the transfection of SINEUP expression vectors (No-insert, SINEUP-SCR and SINEUP-UCHL1) into HEK293T/17 cells. We observed that *Uchl1* mRNAs were mainly localized in the cytoplasm (Figure 10C, c, g, k), whereas the *SINEUP* RNAs were distributed both in the nucleus and the cytoplasm (Figure 10C, f, j). Consistent with the SINEUP-GFP studies, the co-localization of *Uchl1* mRNA and *SINEUP* RNA in the cytoplasm was increased at the SINEUP-UCHL1 transfected cells compared with the SINEUP-SCR transfected cells (Figure 10D). When either PTBP1 (Figure 10E, a1, 2) or HNRNPK (Figure 10E, b1,2) was knocked down, the co-localization of *Uchl1* mRNA and *SINEUP* RNA in the cytoplasm was decreased (Figure 10F, d, h, l, and G), and resulted into the loss of translational up-regulation activity in the UCHL1 protein level compared with siRNA control cells (Figure 10E, c1, 2, SINEUP-UCHL1*, and Figure 10F, d). Note that the *Uchl1* mRNA levels were not changed after the PTBP1 or HNRNPK knockdowns (Supplementary Figure S11). These results suggest that PTBP1 and HNRNPK are important factors of SINEUPs cellular network to enhance translational activity on both exogenous and endogenous target mRNAs.

## DISCUSSION

In this study, we demonstrated that a synthetic *SINEUP* RNA plays a critical role in the enhancement of the translation of its target mRNA by (i) co-localizing with the target mRNA in the cytoplasm, (ii) interacting with SINEUP RBPs to influence the *SINEUP* RNA’s distribution and (iii) increasing target mRNA shifting to the polysome by participating in translational initiation assembly.

By conducting RNA FISH, we revealed that the co-localization of *EGFP* mRNA and *SINEUP-GFP* RNA, and also *Uchl1* mRNA and *SINEUP-UCHL1* RNA in the cytoplasm was required for positive translational regulation. We previously reported that AS-*Uchl1* is enriched in the nucleus and exported to the cytoplasm under rapamycin-induced stress, which inhibits the mTOR (mammalian target of rapamycin) pathway, resulting in up-regulation of UCHL1 translation (9). However, in our subsequent study, the distribution of synthetic *SINEUP* RNAs in the cytoplasm was not increased by inhibition of the mTOR pathway (10). Here, we demonstrated that the synthetic *SINEUP-GFP* RNA localized both in the nucleus and the cytoplasm when *EGFP* mRNA and *SINEUP* RNA co-existed, but was retained in the nucleus in the absence of *EGFP* mRNA. This indicates that synthetic *SINEUP* RNAs may have specific export systems that rely on specific unknown motifs for SINEUP-binding factors, and which differ from the mTOR-dependent export system of AS-*Uchl1*. From loss- and gain-of-function studies of SINEUP RBPs, we found that PTBP1 and HNRNPK proteins have key roles in *SINEUP-GFP* RNA nucleocytoplasmic shuttling and in up-regulating translation.

**Figure 11.**
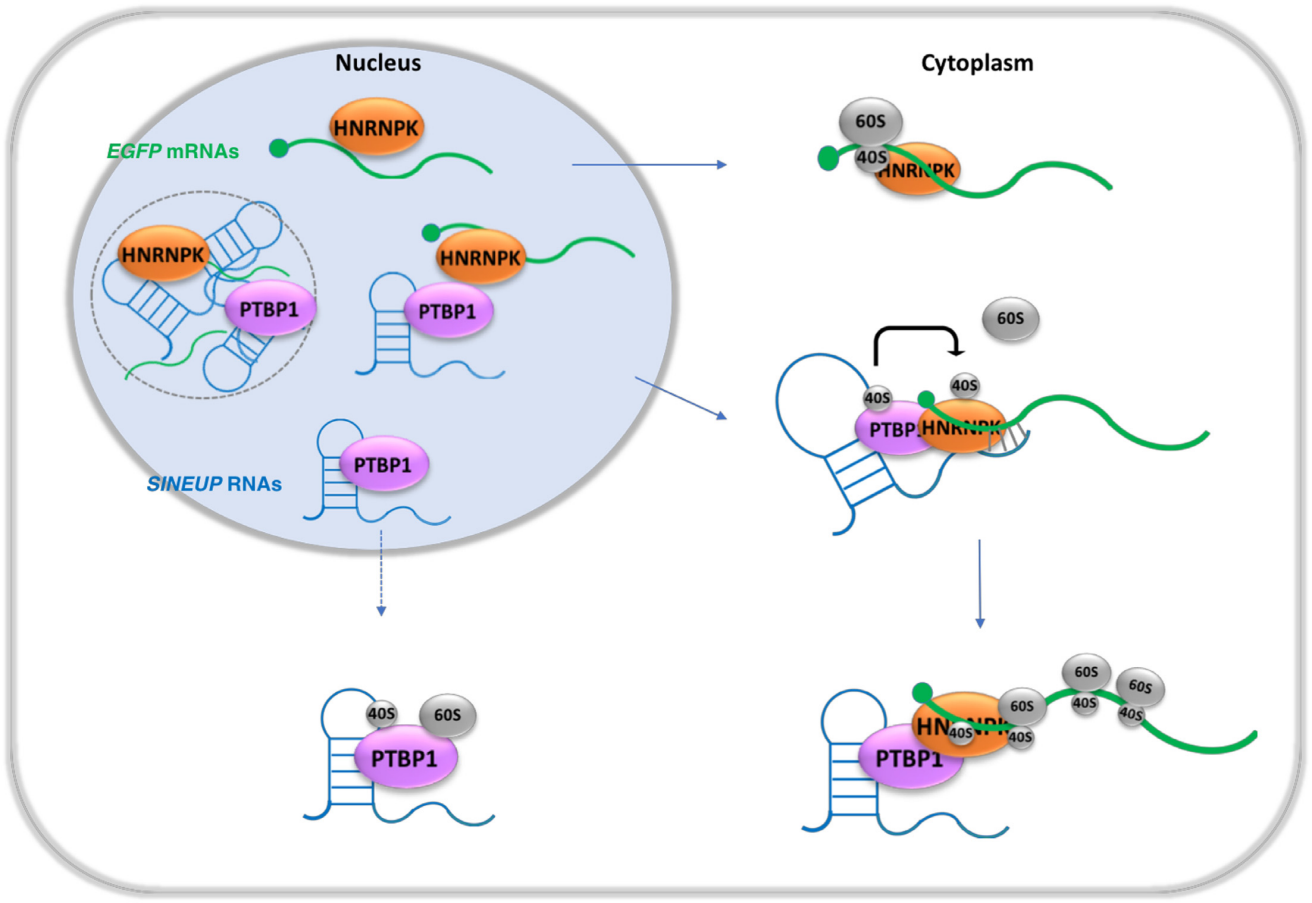
Model of *SINEUP* RNA and SINEUP RBP interactions. SINEUP RBPs (PTBP1 and HNRNPK) participate in *SINEUP* RNA localization both in the nucleus and the cytoplasm. In the nucleus, some RNAs form RNA–protein granules (dotted circle) where immature transcripts likely accumulate to cluster with non-proper conformation, and mature transcripts including *EGFP* mRNA and *SINEUP* RNA form complexes with SINEUP RPBs. These complexes may then be shuttled into the cytoplasm. In the cytoplasm, *SINEUP* RNAs co-operate with the SINEUP RBPs, likely re-modelling *SINEUP* RNA structure, and recruit ribosomal subunits to efficiently supply them to *EGFP* mRNA, resulting in the positive enhancement of *EGFP* mRNA translation. *EGFP* mRNA can be exported into the cytoplasm by itself, but its translation is initiated more efficiently when *SINEUP-GFP* RNA is present in the cytoplasm.

PTBP1 (also known as HNRNPI) is a multifunctional RNA binding protein that participates in alternative splicing, mRNA stabilization, and nucleocytoplasmic shuttling by binding to the polypyrimidine-rich tract in pre-mRNAs (30–32). PTBP1 is known as a binding factor for TOP mRNA, which contains a 5′ terminal oligopyrimidine tract (5′TOP) mostly found in mRNAs encoding ribosomal protein and elongation factors (33,34), and regulates translation of the target TOP mRNA (35) as a *cis*-acting regulator. LARP1, which is known as a 5′TOP mRNA binding protein, stabilizes the target 5′TOP mRNAs by forming complexes with the 40S ribosome subunit (36). In this study, SINEUP-GFP showed direct interaction with 40S, implying that a multimer made up of SINEUP RNA, PTBP1, 40S and the target mRNA may contribute to target mRNA stabilization. There are several reports that PTBP1 is recruited during cap-independent translation to stimulate translational initiation, by re-modelling the target transcripts followed by supplying the small subunit (40S) to binding sites (37,38). In one example of translation initiation, the cricket paralysis virus (CrPV) recruits the 40S ribosome subunit to its RNA with high affinity, and requires eukaryotic elongation factor (eEF)1A and eEF2 to recruit tRNA for assembly of 80S tertiary complexes (39). In this study, several components of ribosomal complexes, such as eEF1A, eEF2 and ribosome proteins, were also detected as SINEUP RBPs (Figure 3). Furthermore, EF1A and PTBP1 were co-sedimented with the RNAs in various fractions ranging from Free/40S-binding RNA to light polysome fractions (Figure 8b, j–m). Together, this indicates that *SINEUP-GFP* RNA, which contains several pyrimidine triplets, may become a scaffold to bind PTBP1, which is predicted to bind pyrimidine triplets (40), and to recruit translational initiation factors. This act likely supplies the 40S subunit to mRNA binding sites, thereby helping initiation assemblies (Figure 11). Although we need to further characterize the protein–protein interactions underpinning the process, our data show that complexes of SINEUP RBPs plus *SINEUP* RNAs affect translational regulation in the initiation phases.

HNRNPK has three KH (K homology) domains, which bind RNAs, and unique nuclear localization signals with bi-directional transport, that enables its export to the nuclear envelope with target mRNAs (27). HNRNPK regulates the target mRNA’s translation positively or negatively, depending on the target mRNA. As an example of positive regulation, HNRNPK bound to *VEGF* mRNA and stimulated the ribosome to bind the mRNA resulting in a shift to heavier polysomes (28). In contrast, in a case of negative regulation, HNRNPK blocked monosome assembly by binding to the 3′ UTR of *c-Src* mRNA thereby repressing the translation (29). In our study, HNRNPK shifted to heavier polysomes with *EGFP* mRNA when HNRNPK was overexpressed and up-regulated *EGFP* mRNA translation, supporting positive regulation; HNRNPK contributed to ribosome assembly only when *EGFP* mRNA and *SINEUP-GFP* RNA co-existed.

When PTBP1 or HNRNPK were overexpressed, EGFP enhancement was observed only when *SINEUP-GFP* RNA and *EGFP* mRNA coexisted, i.e. not when the cells were transfected with EGFP construct alone. Similarly, when PTBP1 was overexpressed, *SINEUP-GFP* RNA distribution was affected only when *SINEUP-GFP* RNA and *EGFP* mRNAs co-existed. These findings imply that the SINEUP RBPs, *SINEUP-GFP* RNA, and *EGFP* mRNAs together form RNA–protein complexes, and that these multiple components are essential for a functional complex. Several studies report protein–protein direct interactions between PTBP1 and HNRNPK, supporting that they function cooperatively in biological processes (41). One lncRNA, *TUNA*, which is known to maintain pluripotency in neuronal cells and forms complexes with PTBP1 and HNRNPK, associates with *Nanog*, *Sox2* and *Fgf4* to activate these pluripotency genes (42). *Lncenc1*, a highly abundant lncRNA in naive embryonic stem cells, recruits PTBP1 and HNRNPK and binds glycolysis genes, thereby regulating cell pluripotency and glycogenesis (43). Our protein–protein interaction experiment using BS3 chemical cross-linked immunoprecipitation (IP) showed that PTBP1 and HNRNPK directly interacted both in the nucleus and the cytoplasm (Supplementary Figure S12A for IP with PTBP1 and S12B for IP with HNRNPK), and that PTBP1 and HNRNPK also directly interacted with 40S and 60S ribosome subunits in the cytoplasm. This suggests that these SINEUP RBPs bind with SINEUP RNAs to form complexes, and participate together in nucleocytoplasmic shuttling and translational regulation. Furthermore, we performed an interaction analysis for *SINEUP-GFP* RNAs and *EGFP* mRNAs using formaldehyde cross-linked immunoprecipitation with J2 antibody, which recognizes double-strand RNAs longer than 40 bp (https://scicons.eu/en/antibodies/j2/). The result showed that *SINEUP-GFP* RNAs and *EGFP* mRNAs formed double strands in the cytoplasm (Supplementary Figure S13). Although further studies are needed to understand the translation regulatory mechanisms of the SINEUP complex as a whole, the formation of RNA–RNA-protein complexes containing *SINEUP-GFP* RNAs, *EGFP* mRNAs and RBPs such as PTBP1 and HNRNPK may be key factors, not only for the nucleocytoplasmic shuttling of the *SINEUP-GFP* RNAs, but also for the recruitment of initiation factors by the translation initiation assembly, to up-regulate translation of the target mRNA in the cytoplasm (Figure 11).

Interestingly, both HNRNPK and PTBP1 are classed as heterogeneous nuclear ribonucleoproteins (RNPs), which mainly participate in alternative mRNA splicing, conformation of RNP assembly to compact transcripts in the nucleus, and nucleocytoplasmic shuttling (44). RNA–RNA intermolecular interactions in RNP granules, which are non-membrane-bound organelles including specific RBPs, contribute to various cellular functions (45). Some components in RNP granules are known to form specific granule assemblies; *MALAT1*, which is a highly abundant lncRNA, localizes in euchromatin loci (46,47), and these nuclear abundant transcripts form nuclear speckles with SC35 (48,49). NONO is known as a core component of paraspeckles in nuclei along with the lncRNA *NEAT1*, and these paraspeckles may relate to transcriptome functional regulation and retention in the nucleus (50–53). ILF3 (Interleukin enhancer-binding factor 3) is known as RNA binding protein to participate in regulation of RNA splicing, stabilization (54,55) and nuclear retention to bind with transposable element in SINEUPs (56). Here, although we observed that some *SINEUP-GFP* RNAs were retained in the nucleus and co-localized with RBPs (Supplementary Figure S2A), we could not characterize these nuclear granules containing *SINEUP* RNAs. This implies that the *SINEUP* RNA and RBPs may form RNA–protein granules that accrue in the nucleus as part of a yet-unknown mechanism for RNA modification and editing. Some reports suggest that nuclear history determines a transcript's fate in the cytoplasm (57,58), and the exon junction complex, which includes splicing factors such as RNPs, affects mRNA destination (59).

Although the binding domain and structural conformation of binding factors in *SINEUP*–protein interactions require further study, our results indicate that intermolecular interactions between *SINEUP* RNAs and the RBPs contribute to the translational up-regulation of the target mRNA. This improves our understanding of the mechanisms of efficient protein translational regulation by functional lncRNAs, and will help to facilitate broad applications of RNA regulation such as nucleic-acid–based therapies.

## DATA AVAILABILITY

The eCLIP-seq data for HNRNPK and PTBP1 discussed in this publication have been deposited in NCBI’s Gene Expression Omnibus (60) and are accessible through GEO Series accession number GSE144345 (https://www.ncbi.nlm.nih.gov/geo/query/acc.cgi?acc=GSE144345).

We have uploaded bigWig files for each of the samples to a UCSC genome browser session which can be accessed using the following link.

https://genome.ucsc.edu/s/mnzvalentine/HNRNPK_PTBP1_eCLIP_hg38_BAM

## Supplementary Material

gkaa814_Supplemental_Files

## Notes

Present address: Ferdous-Ur M. Rahman, Department of Biochemistry and Molecular Genetics, University of Virginia, VA, USA.
