# Supplementary material for: SINEUP long non-coding RNA acts via PTBP1 and HNRNPK to promote translational initiation assemblies
Source: Nucleic Acids Res. 2020 Nov 2;48(20):11626–44. doi: 10.1093/nar/gkaa814 (PMC7672464; doi:10.1093/nar/gkaa814)
Supplement: gkaa814_Supplemental_Files [file gkaa814_supplemental_files.zip › Sup Fig_2021021.pdf]

Supplementary Figure S1

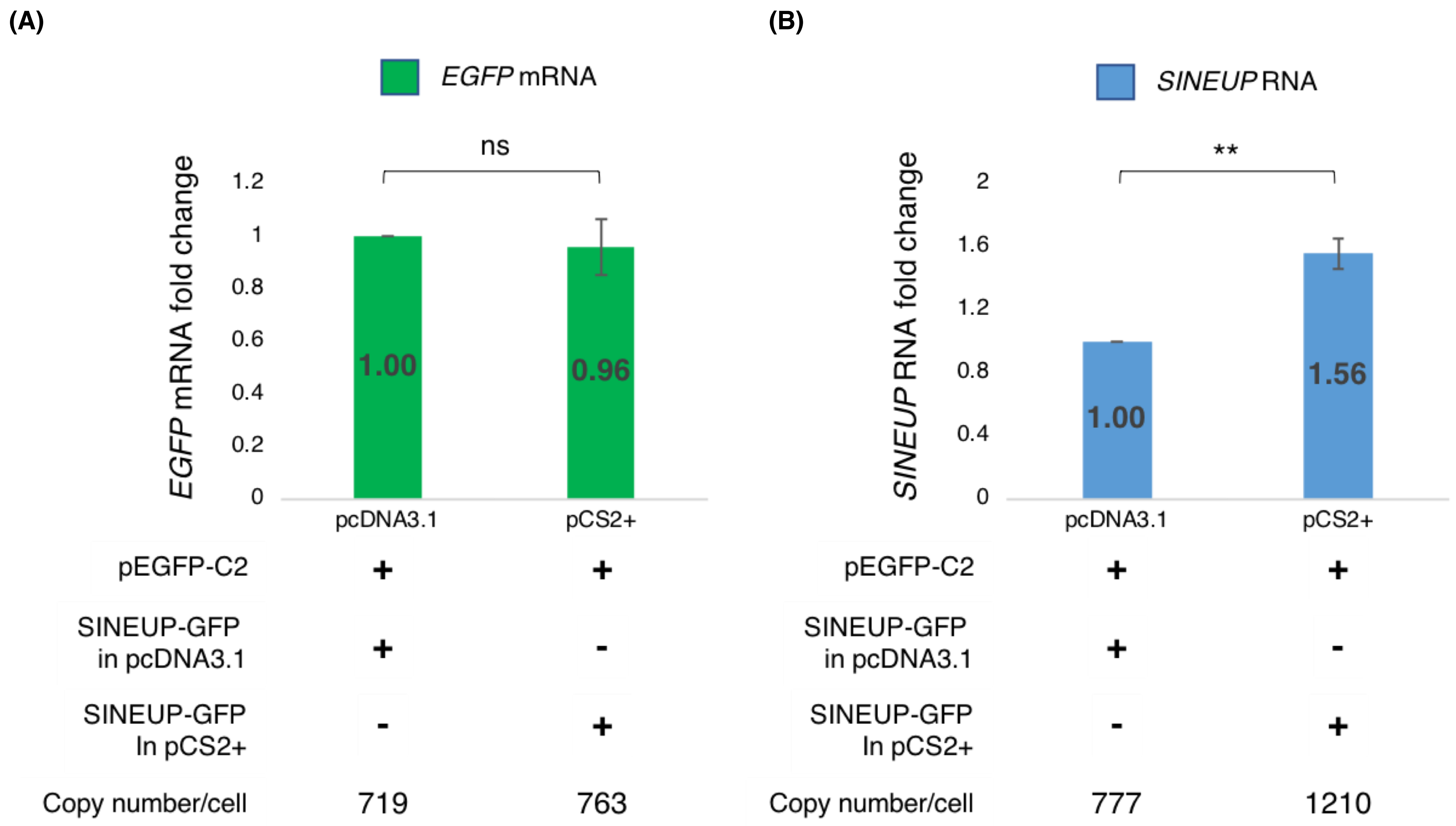

Supplementary Figure S1. Comparison of RNA expression levels in different plasmids for *SINEUP-GFP*.

(A) Quantitative comparison of *EGFP* mRNA levels in cells co-transfected with EGFP vector and SINEUP-GFP in either a pcDNA3.1 or pCS2+ vector. Numbers in the bottom row indicate mean transcript copy number per cell quantified by absolute qPCR quantification. ns: not significant by Student's *t*-test. Data are means ± SD of at least 3 independent experiments.

(B) Quantitative comparison of *SINEUP-GFP* RNA levels in cells co-transfected with EGFP vector and SINRUP-GFP in either a pcDNA3.1 or pCS2+ vector. Numbers in the bottom row indicate mean transcript copy number per cell quantified by absolute qPCR quantification. **p < 0.01 by Student's *t*-test. Data are means ± SD of at least 3 independent experiments.

Supplementary Figure S2

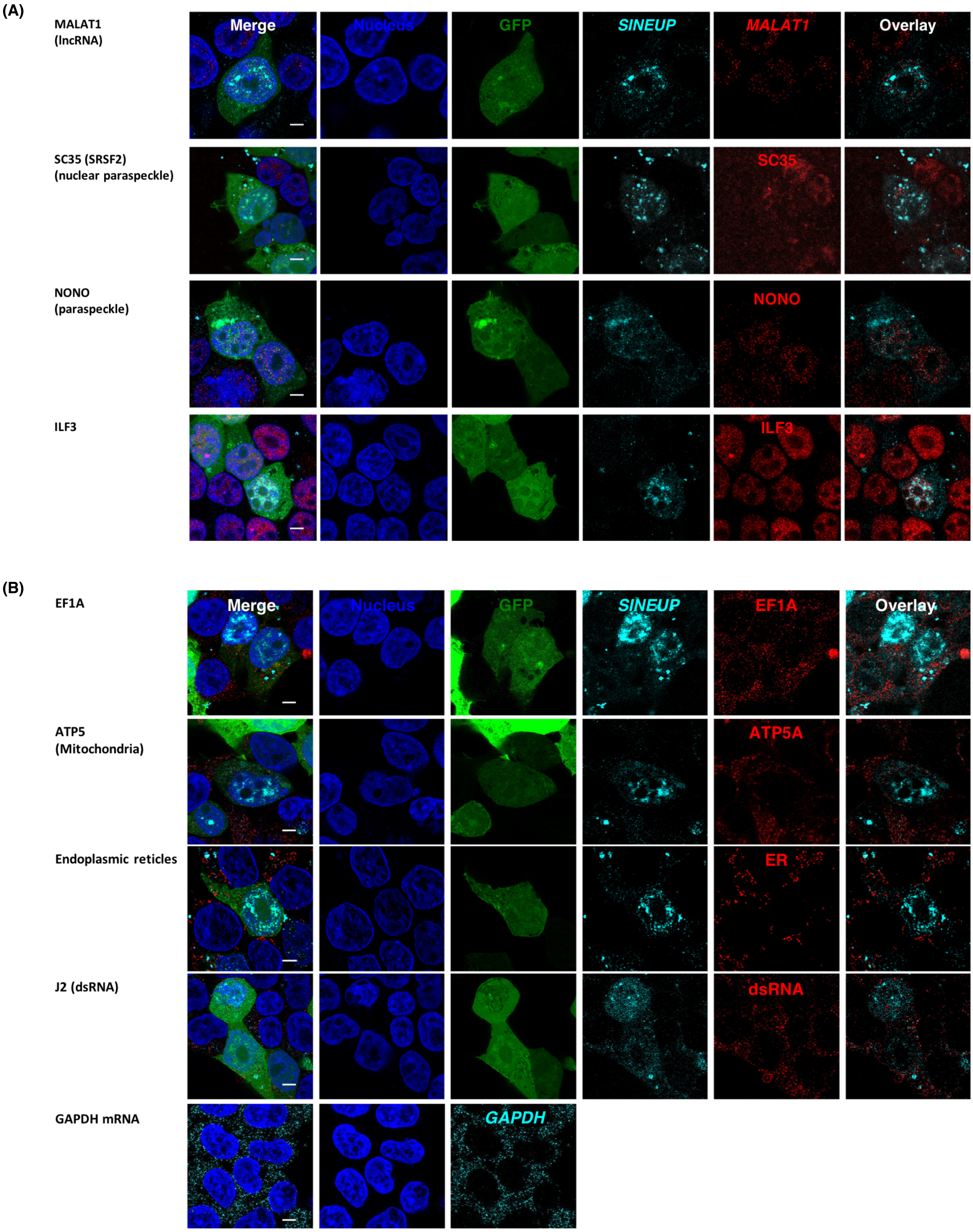

Supplementary Figure S2. Co-localization of *SINEUP-GFP* RNAs with organelle markers.
(A) Immunofluorescence of nuclear organelle markers, and RNA FISH for *MALAT1* RNA. Bars indicate 5 μm.
(B) Immunofluorescence of cytoplasmic protein markers, dsRNA and RNA FISH for *GAPDH* mRNA. Bars indicate 5 μm.

Supplementary Figure S3

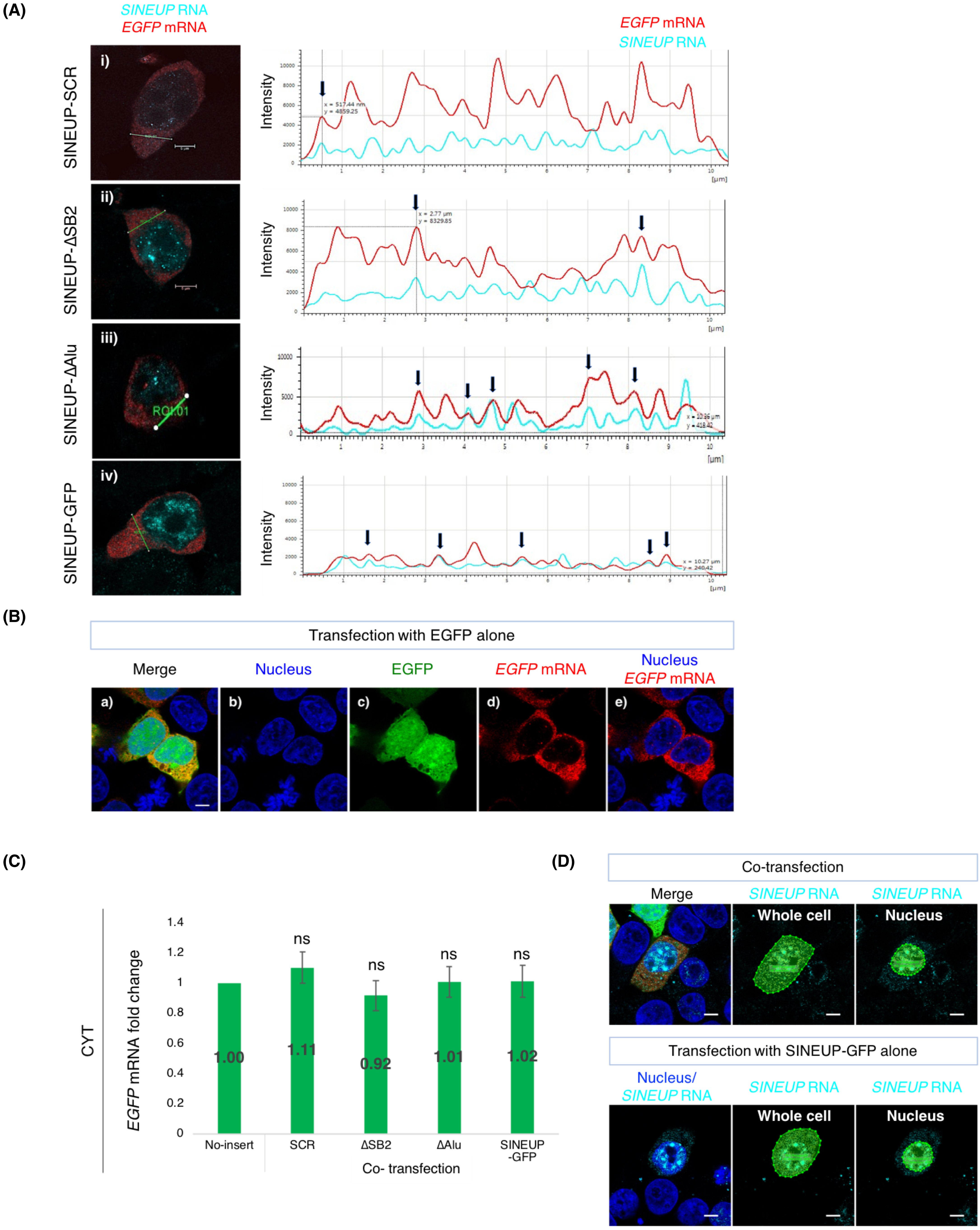

Supplementary Figure S3. Detection of mutant *SINEUP* RNA signals

(A) RNA intensity images. The graphs show RNA signal intensity in the region of interest (ROI), indicated by the line on the corresponding images on the left. The X axis shows distance along this ROI line, and the Y axis shows signal intensity. Arrows indicate peaks where *EGFP* mRNA and *SINEUP* RNA signals overlap.

(B) Subcellular localization of *EGFP* mRNA following transfection with EGFP expression vectors alone. Bars indicate 5 µm.

(C) Cytoplasmic expression level of *EGFP* mRNA among the mutant constructs. ns: not significant by Student's *t*-test. Data are means ± SD of at least 3 independent experiments.

(D) Spot detected images in cells after co-transfection, and transfection with SINEUP-GFP alone. Using icy Spot Detector, signals were detected from both the whole cell and the nucleus. Bars indicate 5 µm.

Supplementary Figure S4

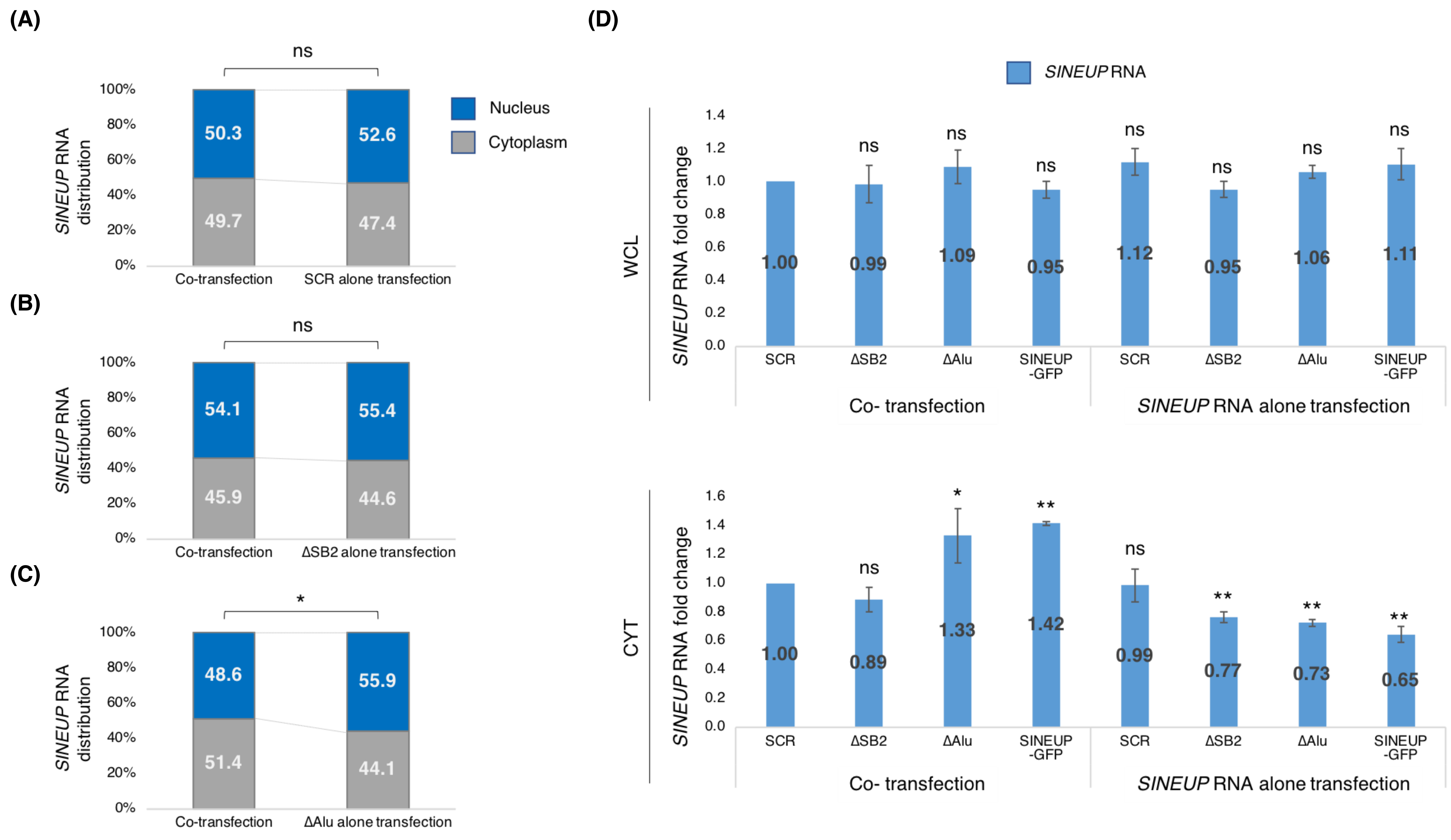

Supplementary Figure S4. Comparison of subcellular distribution of SINEUP mutants in cells transfected with *SINEUP* RNA vector alone and cells co-transfected with EGFP and *SINEUP* RNA vectors

(A-C) Nuclear and cytoplasmic distributions of the SINEUP mutants SINEUP-SCR (A), SINEUP-ΔSB2 (B) and SINEUP-ΔAlu (C) were compared with and without transfection of EGFP vector. *p < 0.05, ns: not significant by Student's *t*-test. Data were collected from at least 10 independent cell images.

(D) Quantification of the *SINEUP* RNA levels in the cytoplasmic fraction compared in the presence and absence of *EGFP* mRNA. *p <0.05, **p < 0.01, ns: not significant by Student's *t*-test. Data are means ± SD from at least 3 independent experiments. WCL indicates total RNAs from the whole cell lysate, and CYT indicates cytoplasmic RNAs from the cytoplasmic fraction.

Supplementary Figure S5

(A)

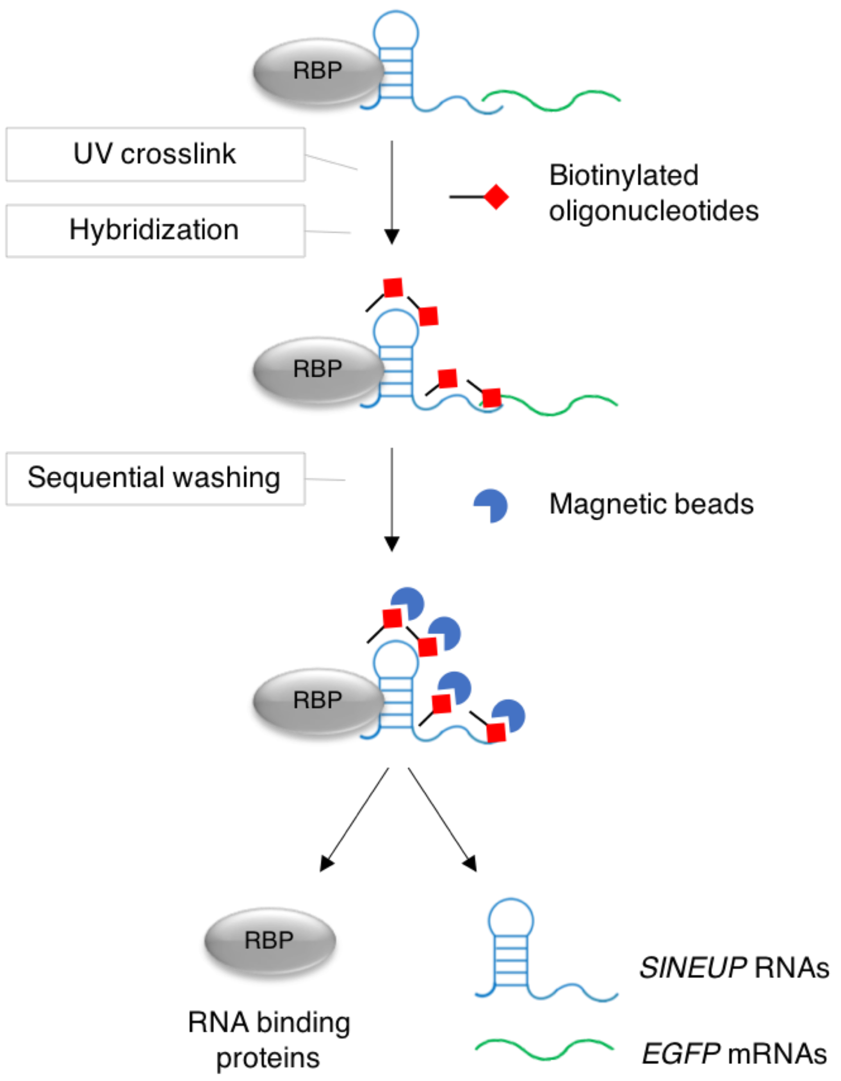

(B)

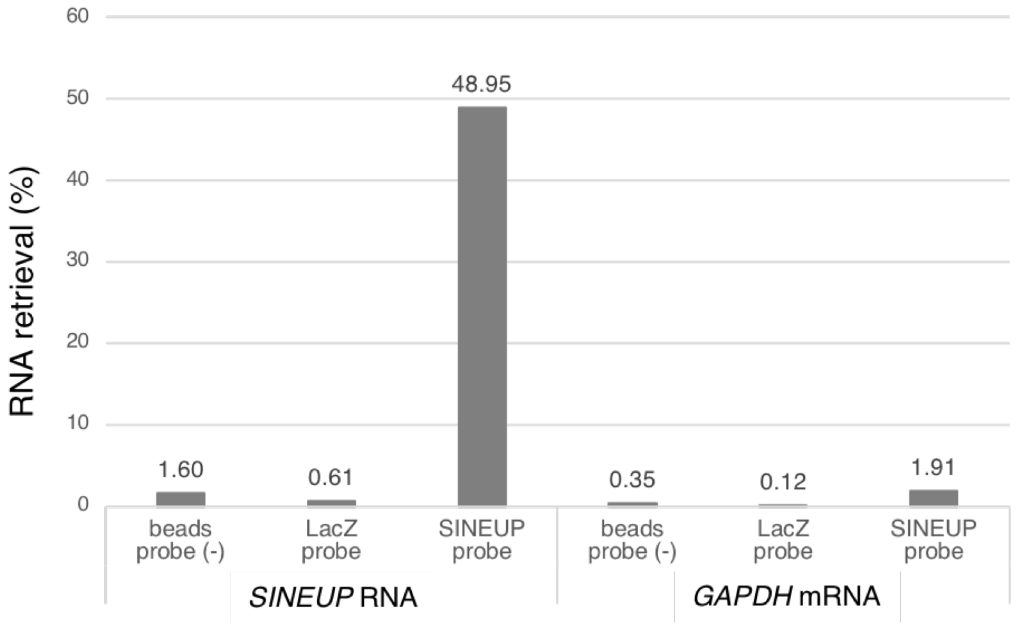

(C)

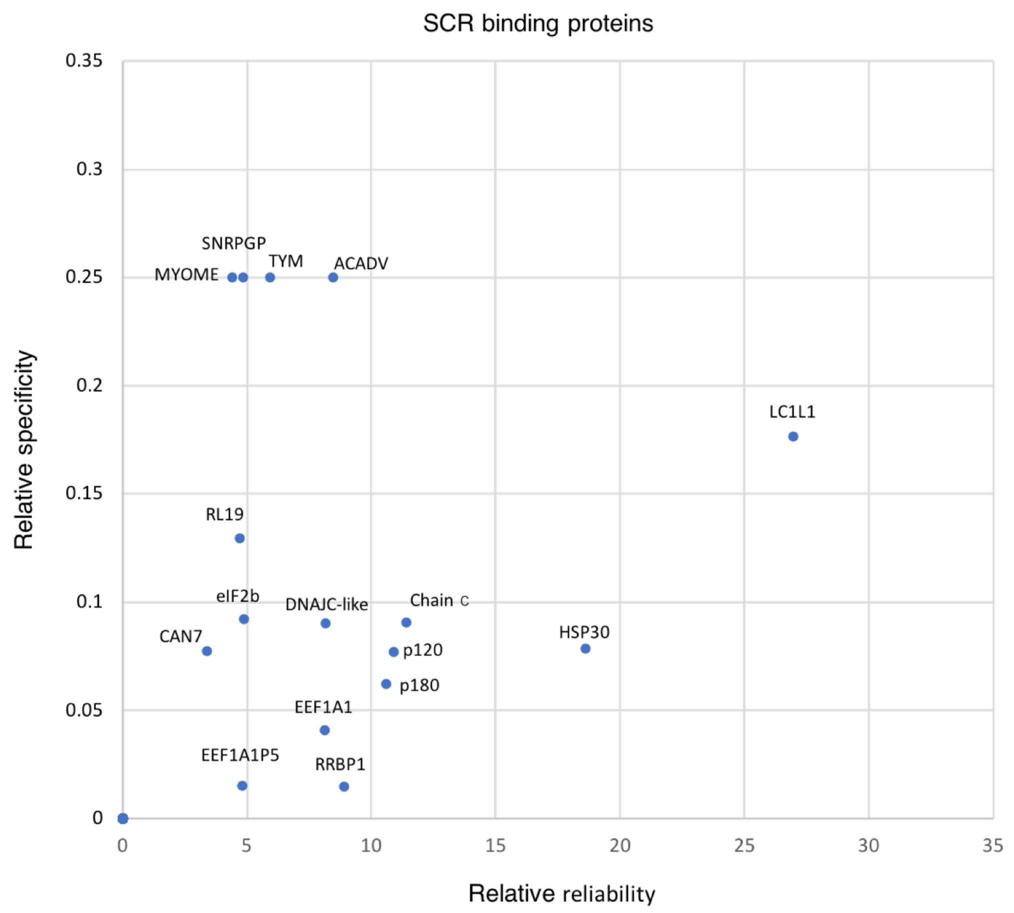

(D)

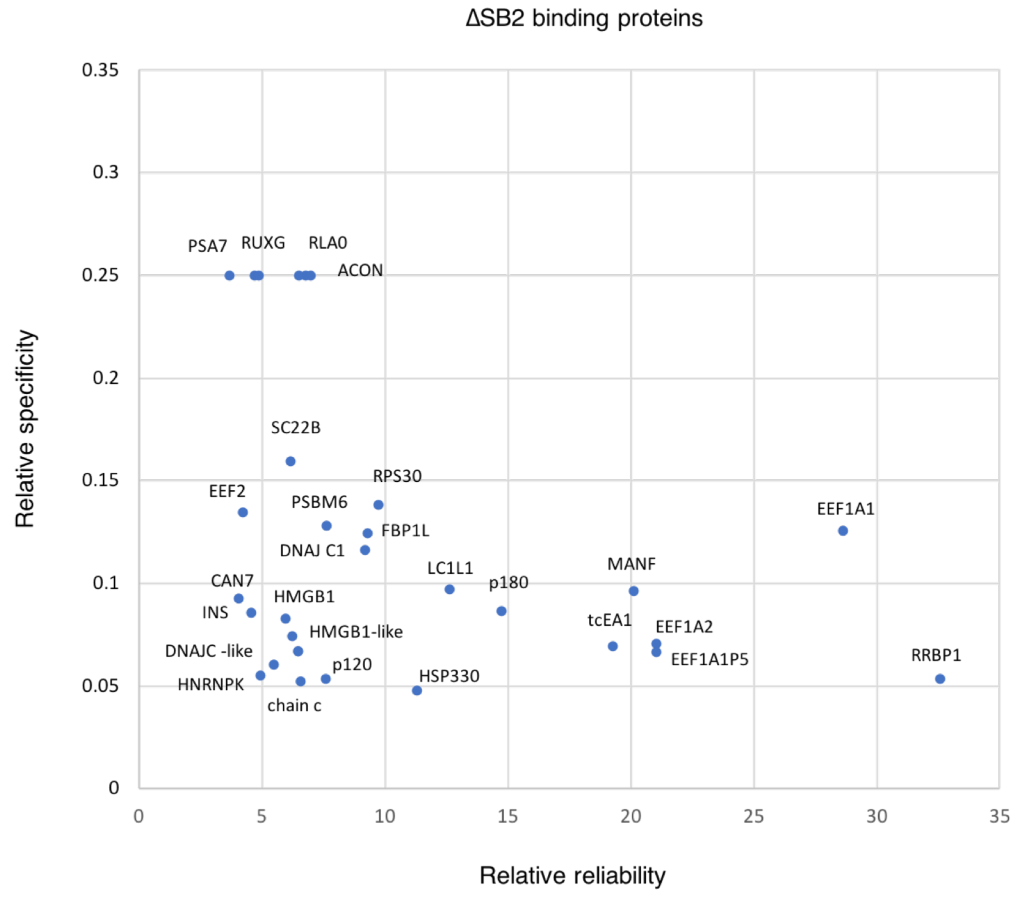

(E)

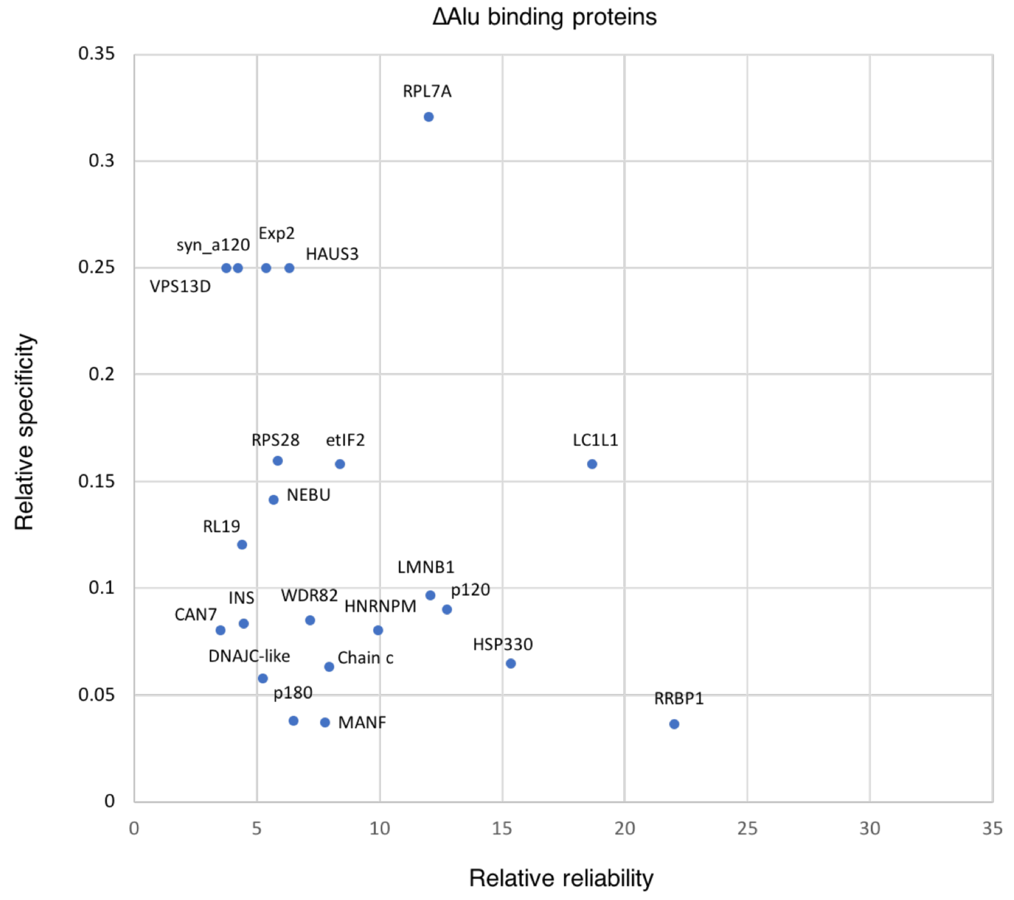

Supplementary Figure S5. SINEUP RNA-protein interaction

(A) Schematic workflow of the modified ChIRP protocol (Chu, *et al*, 2015) (15).

(B) Percentage retrieval of RNA by modified ChIRP with SINEUP RNA probe. Biotinylated SINEUP-GFP probe specifically enriched *SINEUP-GFP* RNA compared with the Magna ChIRP negative control probe (LacZ) and magnetic beads alone (probe (-)). *GAPDH* mRNA was tested as a negative control to assess non-specific interaction.

(C-E) SINEUP-SCR (C), SINEUP-ΔSB2 (D), and SINEUP-ΔAlu (E) RBPs plotted according to relative reliability and specificity.

Supplementary Figure S6

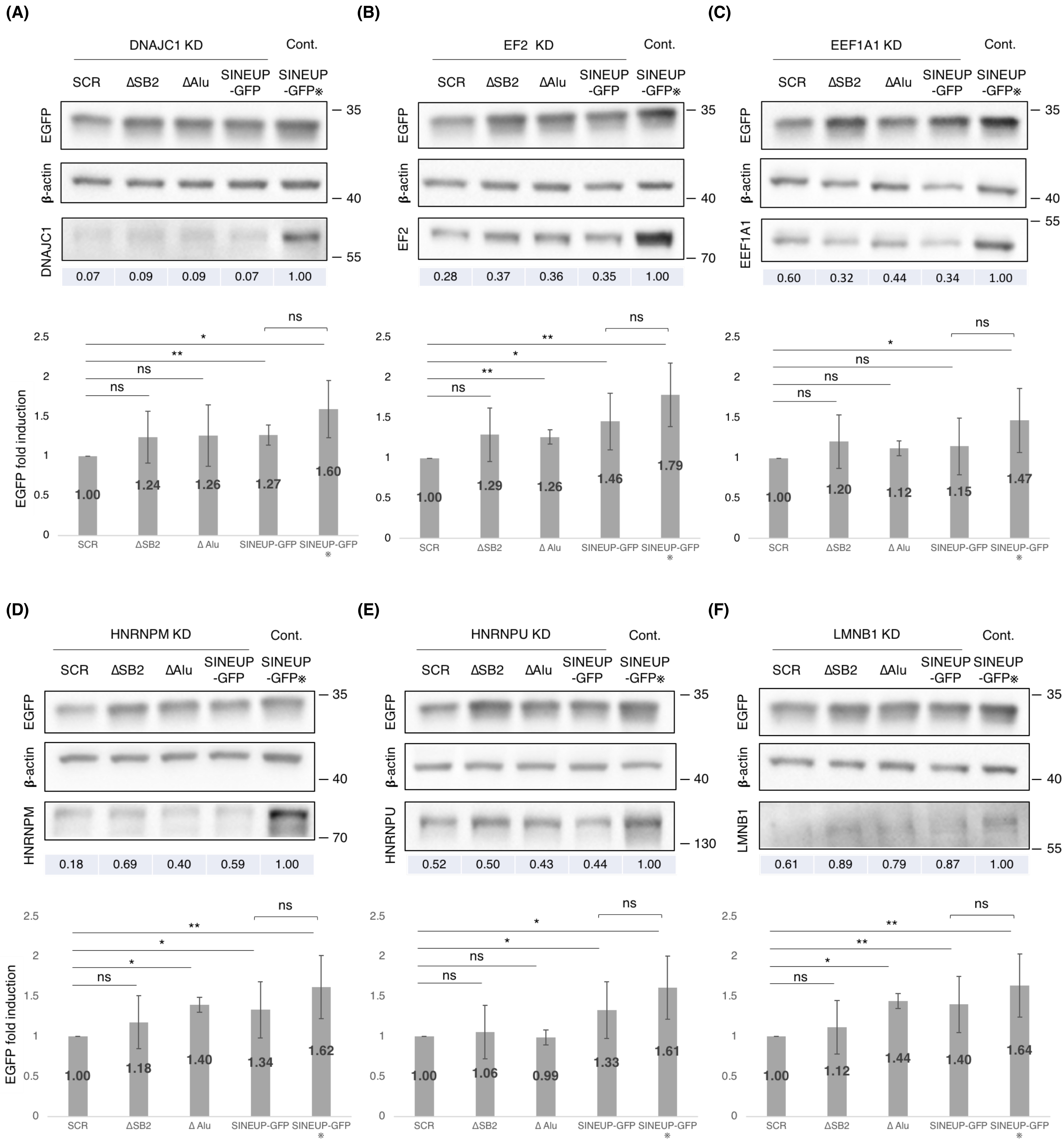

Supplementary Figure S6. Knockdown of SINEUP RBPs.

(A–F) Knockdown of SINEUP RBPs. Numbers in the bottom row indicate knockdown efficiency compared with cells co-transfected with the SINEUP-GFP vector and negative control siRNA (SINEUP-GFP※ in Fig 4C1 and C2). $^{*}p < 0.05$, $^{**}p < 0.01$, ns: not significant by Student's *t*-test. Data are means ± SD of at least 3 independent experiments.

Supplementary Figure S7

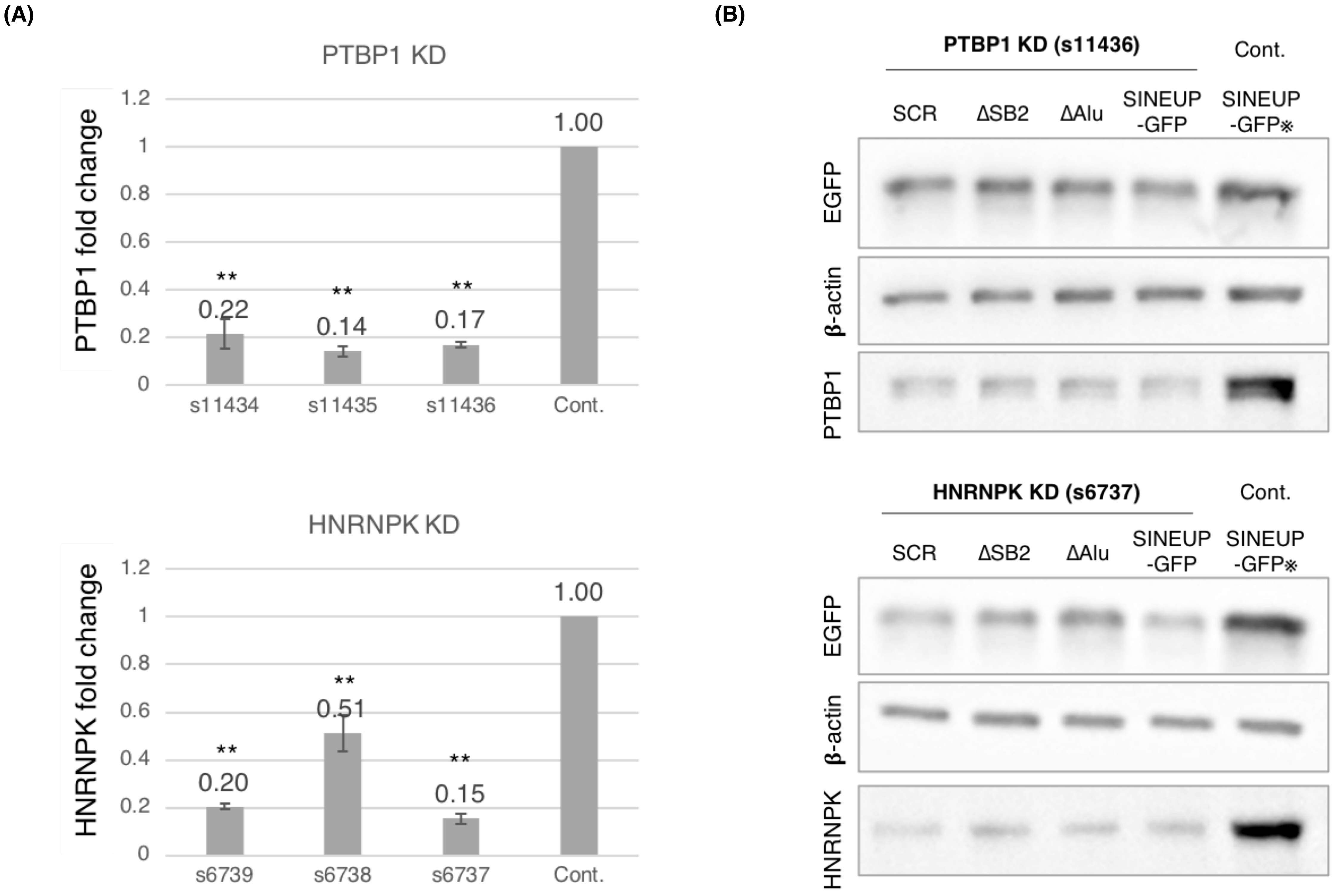

Supplementary Figure S7. Knockdown by siRNAs targeting different regions of the RBPs.

(A) Knockdown efficiency by several siRNAs of PTBP1 and HNRNPK compared with a SINEUP-GFP transfected control siRNA (SINEUP-GFP※). **$p < 0.01$ by Student's *t*-test. Data are means ± SD from at least 3 independent experiments.

(B) Representative Western blotting images showing knockdowns of domain mutant SINEUPs by a PTBP1 (s11436), and HNRNPK (s6737) siRNA.

Supplementary Figure S8

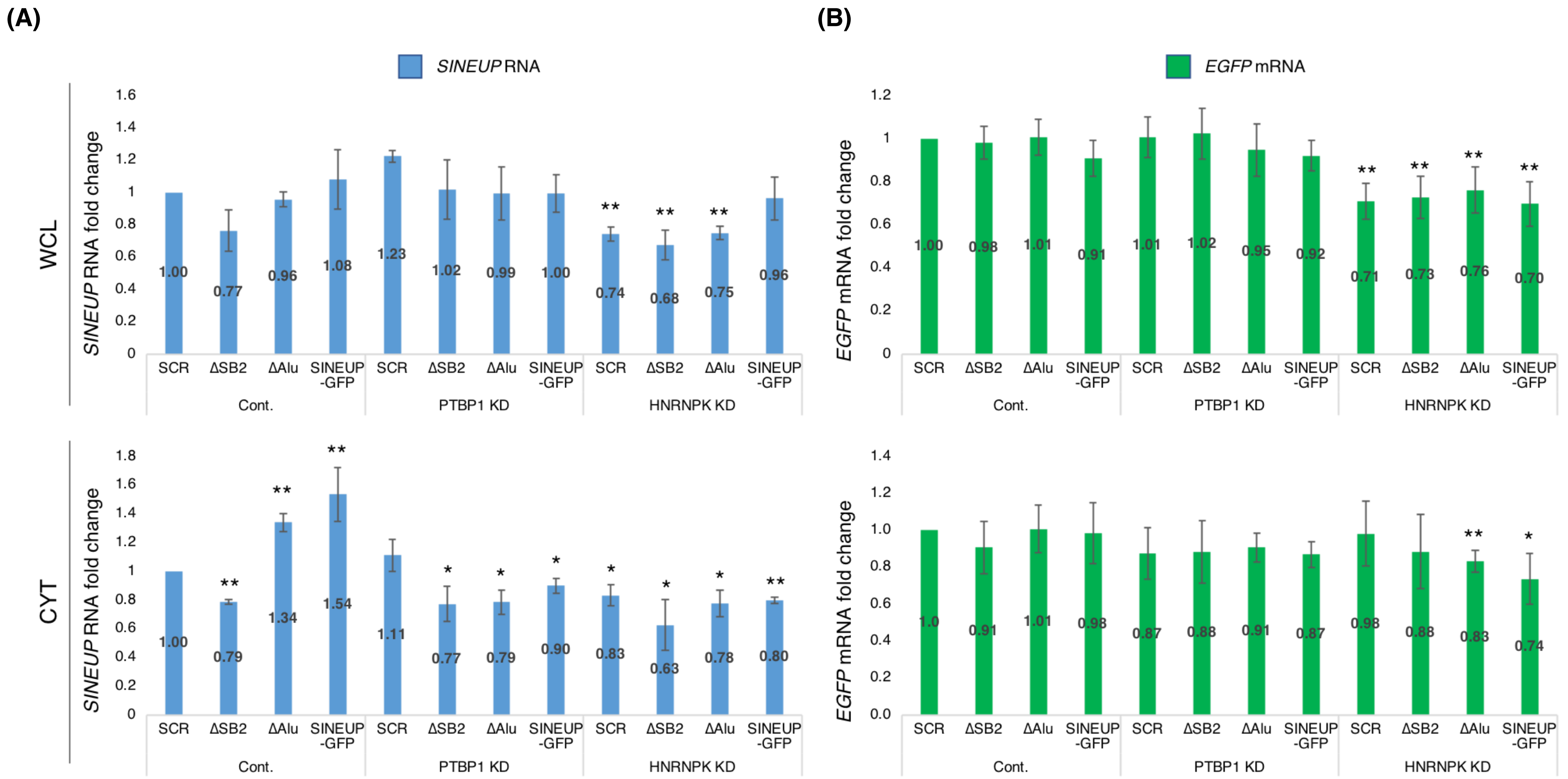

Supplementary Figure S8. Quantification of RNA levels when PTBP1 or HNRNPK were knocked down.
(A, B) Quantitative comparison of *SINEUP* RNA (A) and *EGFP* mRNA (B) expression levels between cells after knockdown (KD) of PTBP1 or HNRNPK, and non-knockdown of these proteins (Cont.). *p < 0.05, **p < 0.01 by Student's *t*-test. Data are means ± SD from at least 3 independent experiments. WCL indicates total RNAs from the whole cell lysate, and CYT indicates cytoplasmic RNAs from the cytoplasmic fraction.

Supplementary Figure S9

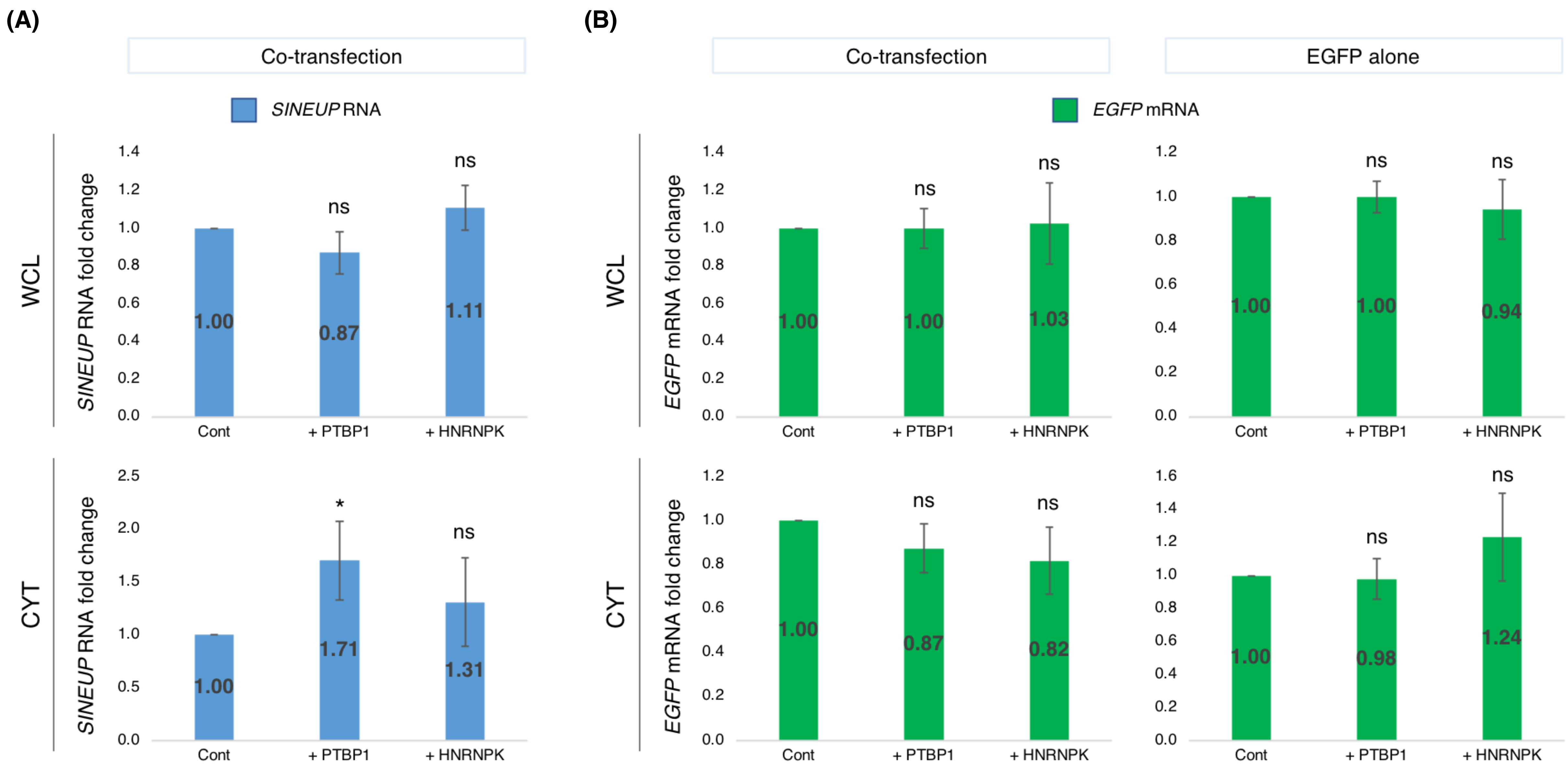

Supplementary Figure S9. Quantification of RNA levels when PTBP1 or HNRNPK were overexpressed.
(A, B) Quantitative comparison of *SINEUP* RNA (A) and *EGFP* mRNA (B) expression levels between cells overexpressing PTBP1 or HNRNPK, and non-overexpression of these proteins (Cont.). *p < 0.05, ns: not significant by Student's *t*-test. Data are means ± SD from at least 3 independent experiments. WCL indicates total RNAs from the whole cell lysate, and CYT indicates cytoplasmic RNAs from the cytoplasmic fraction.

Supplementary Figure S10

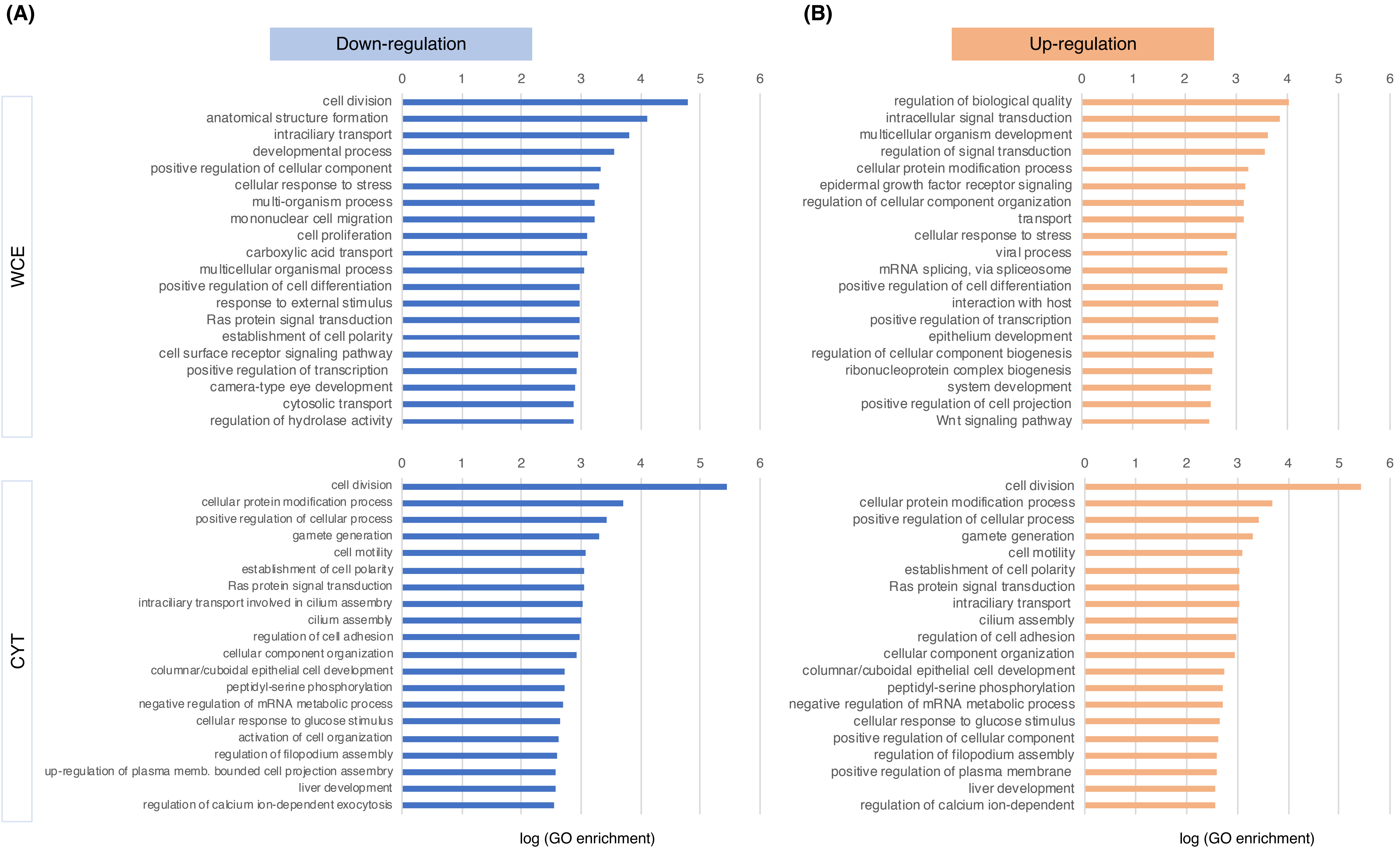

Supplementary Figure S10. Gene Ontology (GO) analysis by knockdown of HNRNPK
(A-B) Top twenty enriched down-regulated (A) and up-regulated (B) Biological Process (BP) GO terms following knockdown of HNRNPK in the whole cell extract (WCE) and cytoplasmic (CYT) fractions in MCF7 cell.

Supplementary Figure S11

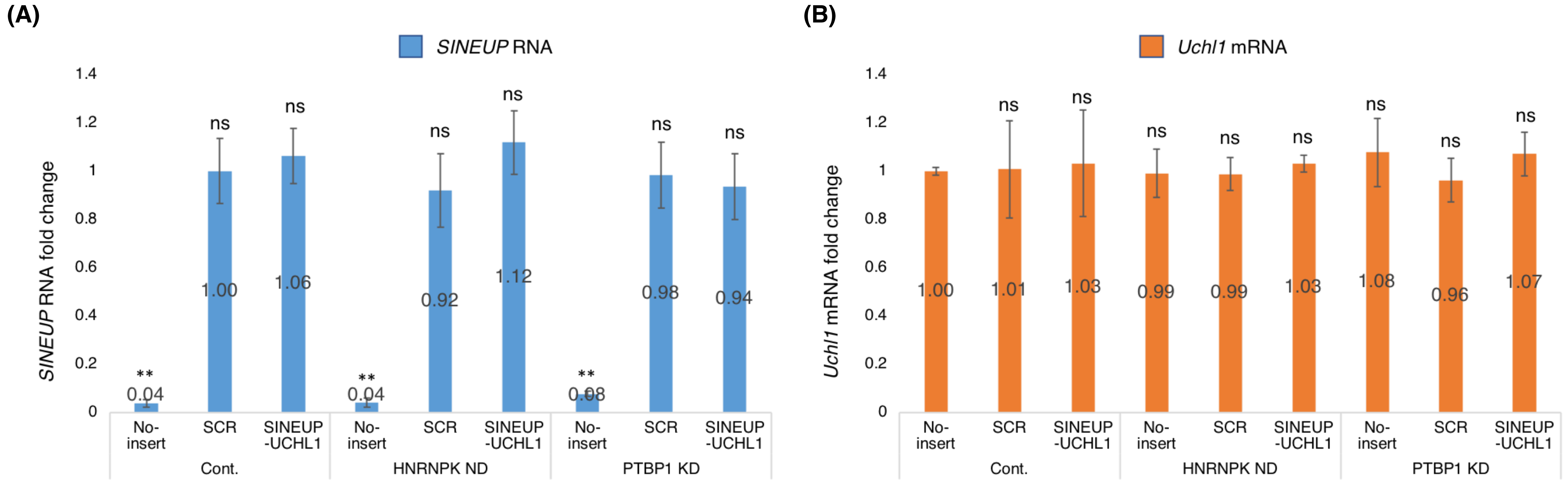

Supplementary Figure S11. Quantification of RNA levels when PTBP1 or HNRNPK were knocked down.
(A, B) Quantitative comparison of *SINEUP* RNA (A) and *Uchl1* mRNA (B) expression levels between cells after knockdown (KD) of PTBP1 or HNRNPK, and non-knockdown of these proteins (Cont.). **p < 0.01, ns: not significant, by Student's *t*-test. Data are means ± SD from at least 3 independent experiments.

Supplementary Figure S12

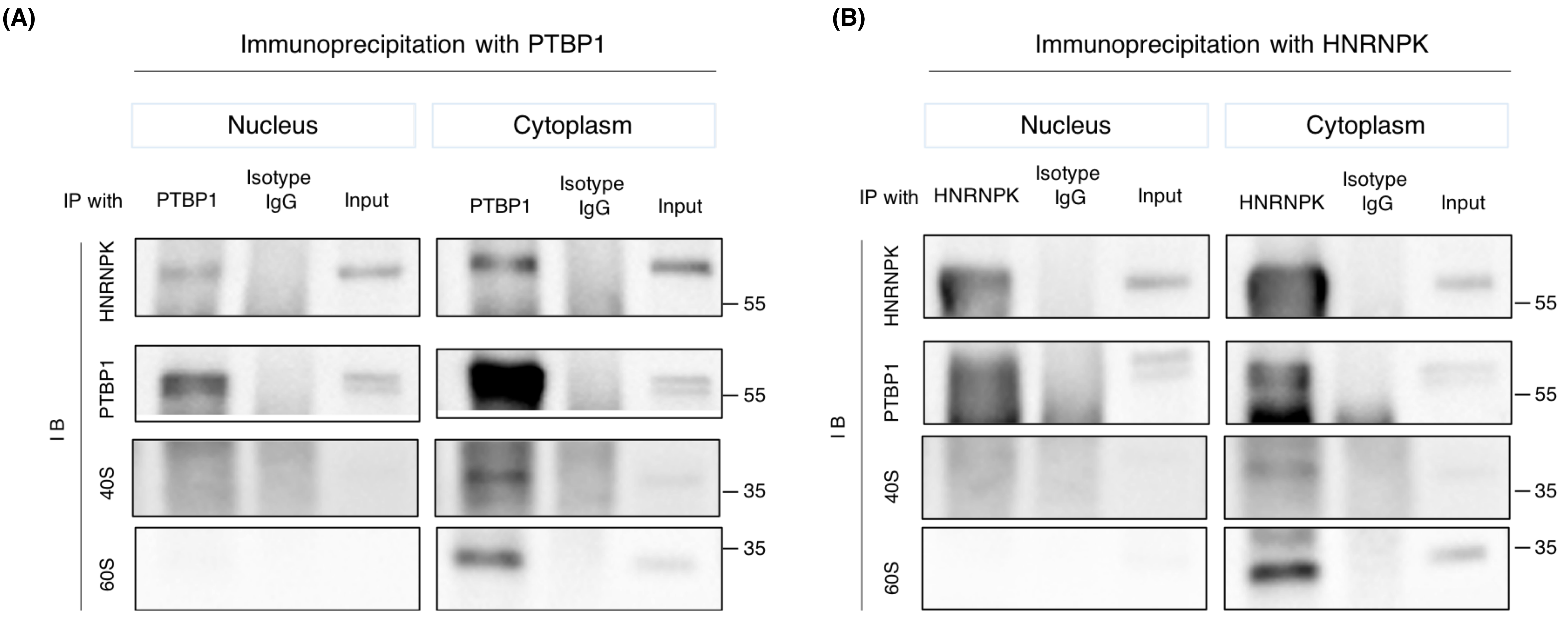

Supplementary Figure S12. Protein–protein direct interaction by chemical crosslinking with bis (sulfosuccinimidyl) suberate (BS3). (A-B) Representative Western blotting images of immunoprecipitation (IP) with PTBP1 antibody (A) or HNRNPK antibody (B). Isotype IgG was used as the negative antibody control. IP products were detected by immunoblotting (IB) each protein antibody.

Supplementary Figure S13

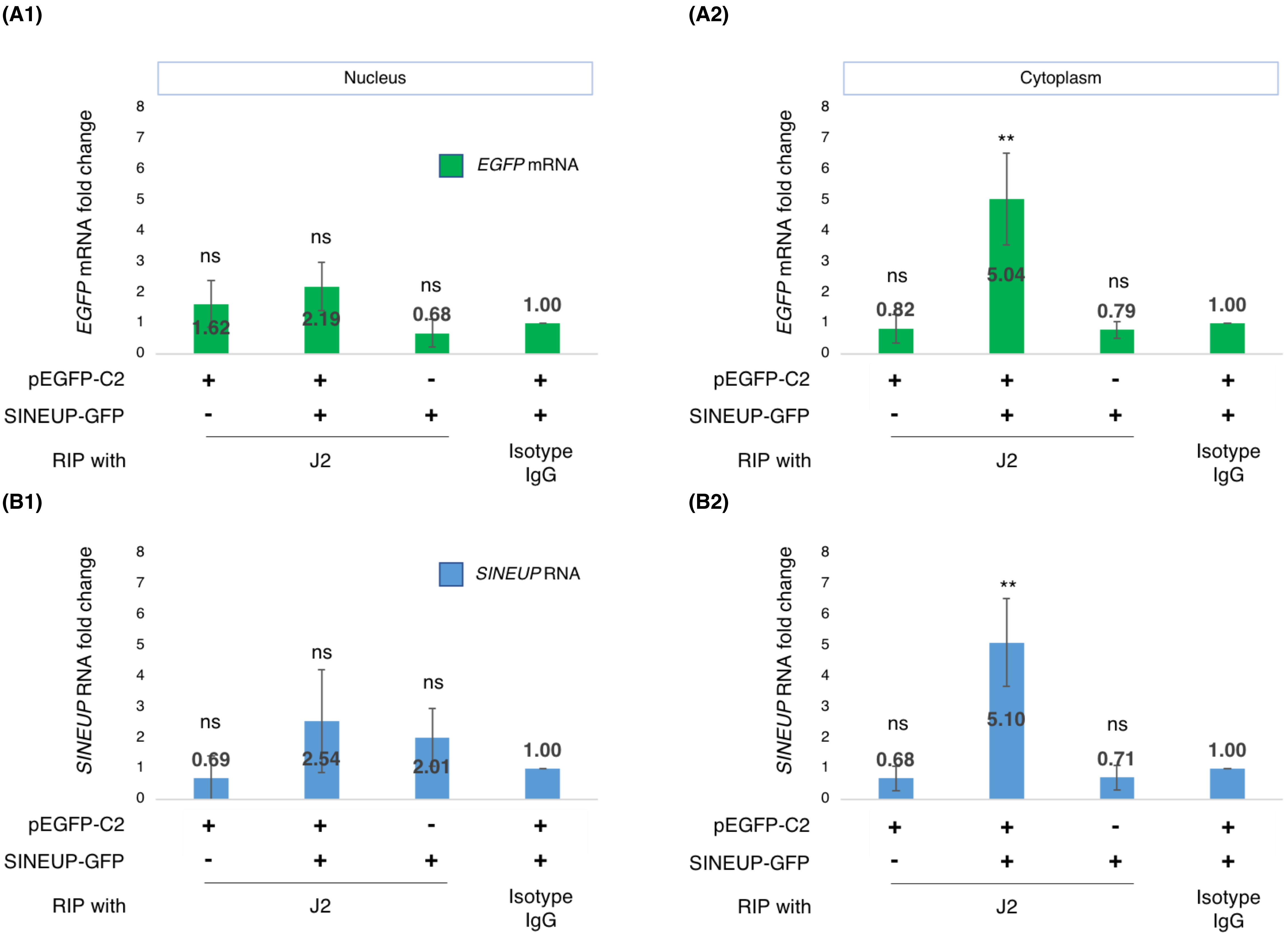

Supplementary Figure S13. RNA immunoprecipitation pulled down with dsRNAs.

(A) *EGFP* mRNA immunoprecipitation with J2 antibody in the nucleus (A1) and cytoplasm (A2). Isotype IgG was used as the negative antibody control. ns: not significant by Student's *t*-test. Data are means ± SD of at least 3 independent experiments.

(B) *SINEUP-GFP* RNA immunoprecipitation with J2 antibody in the nucleus (B1) and cytoplasm (B2). Isotype IgG was used as the negative antibody control. **p < 0.01, ns: not significant, by Student's *t*-test. Data are means ± SD from at least 3 independent experiments.
